# *C9orf72*-associated and sporadic FTD patient iPSC-microglia show differences in phagocytosis and gene expression

**DOI:** 10.1016/j.stemcr.2026.103026

**Published:** 2026-08-06

**Authors:** Hannah Rostalski, Tomi Hietanen, Dorit Hoffmann, Sami Heikkinen, Nadine Huber, Ashutosh Dhingra, Salvador Rodriguez-Nieto, Teemu Kuulasmaa, Sohvi Ohtonen, Henna Jäntti, Viivi Pekkala, Stina Leskelä, Petra Mäkinen, Kasper Katisko, Päivi Hartikainen, Šárka Lehtonen, Eino Solje, Jari Koistinaho, Tarja Malm, Anne M. Portaankorva, Teemu Natunen, Henna Martiskainen, Mari Takalo, Mikko Hiltunen, Annakaisa Haapasalo

**Affiliations:** 1 A.I. Virtanen Institute for Molecular Sciences, University of Eastern Finland, Kuopio, Finland; 2 Institute of Biomedicine, University of Eastern Finland, Kuopio, Finland; 3 German Center for Neurodegenerative Diseases (DZNE), Tübingen, Germany; 4 Institute of Clinical Medicine - Neurology, University of Eastern Finland, Kuopio, Finland; 5 Neuro Center, Neurology, Kuopio University Hospital, Kuopio, Finland; 6 Neuroscience Center, Helsinki Institute of Life Science (HiLIFE), University of Helsinki, Helsinki, Finland; 7 Department of Neurology, University of Helsinki, Helsinki, Finland

**Keywords:** ALS, FTD, *C9orf72* hexanucleotide repeat expansion, microglia, phagocytosis, RNA sequencing

## Abstract

*C9orf72* hexanucleotide repeat expansion (C9-HRE) is a major genetic cause of amyotrophic lateral sclerosis and frontotemporal dementia (FTD). However, approximately half of the FTD patients are sporadic without a clear genetic background. To compare characteristics of microglia from different FTD subtypes, we generated induced pluripotent stem cell-derived microglia (iMG) from sporadic and C9-HRE-carrying behavioral variant FTD (bvFTD) patients and healthy controls. C9-HRE iMG displayed C9-HRE-associated RNA foci and dipeptide repeat proteins. All bvFTD iMG had fewer LAMP2-A-positive vesicles compared to control iMG. Additionally, C9-HRE iMG showed significantly increased LC3BII/I conversion after bafilomycin A1 treatment and altered phagocytic activity. The gene expression profile of C9-HRE iMG only modestly differed from the control iMG, but was greatly different from the sporadic bvFTD patient iMG. Our data show alterations in phagocytic and autophagosomal/lysosomal pathways and gene expression profiles between C9-HRE and sporadic bvFTD iMG for the first time.

## Highlights

• iPSC-derived microglia from sporadic *vs* C9-HRE bvFTD patients were compared for the first time
• bvFTD iPSC-microglia recapitulate main C9-HRE-related pathological hallmarks
• Phagocytosis and autophagosomal/lysosomal pathways are changed in bvFTD microglia
• C9-HRE and sporadic microglia show differing gene expression profiles

## Teaser

Rostalski and Hietanen and colleagues found that induced pluripotent stem cell-derived microglia (iMG) from C9-HRE-carrying and sporadic bvFTD patients show differential changes related to gene expression, phagocytosis, and autophagosomal/lysosomal pathways. This study provides new insights for the first time into common and differing phenotypes and function between the iMG from C9-HRE-carrying and sporadic bvFTD patients.

## Introduction

Microglia perform key tasks during brain development, homeostasis, aging and disease (De Schepper et al., 2021; Fujita and Yamashita, 2021; Spittau, 2017; Wake et al., 2011). Since several risk genes for neurodegenerative diseases (including *C9orf72*) are expressed by microglia (Haenseler et al., 2017; Zhang et al., 2016), studying microglia-dependent disease mechanisms is important.

Frontotemporal dementia (FTD) and amyotrophic lateral sclerosis (ALS) are neurodegenerative diseases within the same neuropathological and genetic disease spectrum but with distinct clinical phenotypes. Whereas FTD predominantly affects frontal and temporal regions of the brain and manifests by behavioral or linguistic changes, patients with ALS suffer from motor neuron degeneration (Kiernan et al., 2011; Rabinovici and Miller, 2010; Rascovsky et al., 2011). FTD and ALS can share an overlapping causative genetic background, including the *C9orf72* hexanucleotide repeat expansion (C9-HRE) (DeJesus-Hernandez et al., 2011; Majounie et al., 2012; Renton et al., 2011), and are often characterized by similar central nervous system pathologies, e.g., TDP-43 accumulation (Neumann et al., 2009, 2020), dysfunction in protein degradation pathways, including autophagy, accumulation of misfolded and toxic protein aggregates, neuronal death, and glial cell activation (Banerjee et al., 2023; Menzies et al., 2015).

In this study, we aimed to characterize cell pathological and functional phenotypes of microglia from FTD patients. To this end, we generated induced pluripotent stem cell (iPSC)-derived microglia (iMG) from healthy controls and C9-HRE-carrying and sporadic patients clinically diagnosed with behavioral variant FTD (bvFTD) for the first time. We found that iMG of C9-HRE carriers showed RNA foci and dipeptide repeat (DPR) proteins but no signs of *C9orf72* haploinsufficiency or TDP-43 pathology. Interestingly, bvFTD iMG showed lysosomal changes and significantly altered phagocytic activity, especially in the presence of the C9-HRE. Global mRNA sequencing revealed differential expression of genes related to RNA, protein, and mitochondrial metabolism pathways in C9-HRE iMG compared to the sporadic bvFTD iMG. Compared to healthy control iMG, C9-HRE iMG showed enrichment in genes related to acute inflammatory response. The present study, by comparing C9-HRE and sporadic bvFTD iMG to healthy controls, provides completely new insights into differences in gene expression and cellular functions in these patient-derived immune cells.

## Results

### iMG of C9-HRE bvFTD patients show typical C9-HRE-related pathological hallmarks

**Figure 1:**
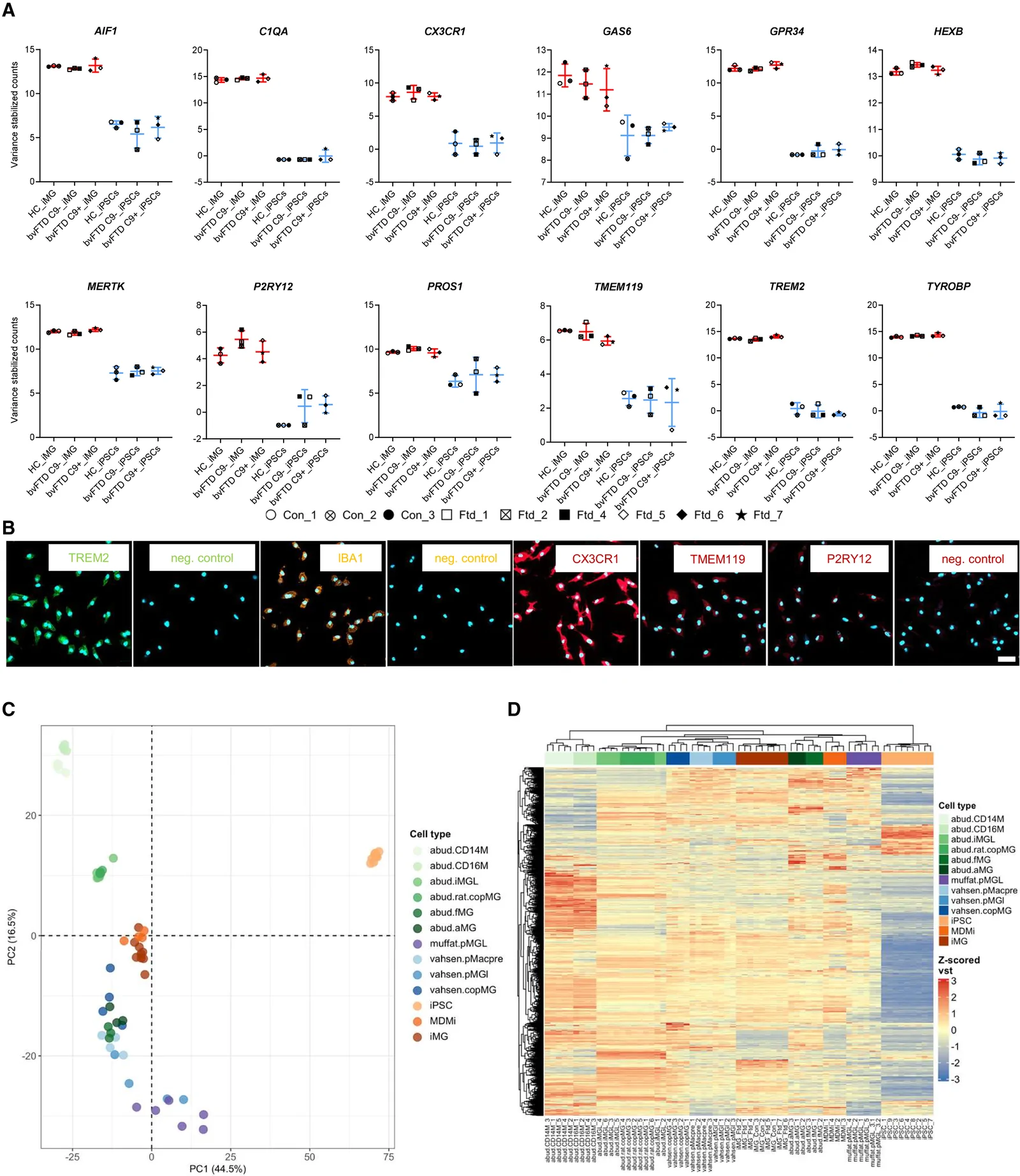
Microglial identity of iMG from healthy controls, sporadic (C9–) and C9-HRE-carrying (C9+) bvFTD patients (A) RNA sequencing data for each donor, displayed as mean ± standard deviation of variance stabilized counts, show expression of selected microglial genes in iMG (red) vs. iPSCs (blue); each data point represents one RNA sample per donor. The individual iPSC and iMG lines are indicated by different symbols below the plots. (B) iMG of a healthy donor (Con_1) were stained for selected microglial marker proteins. The corresponding negative control stainings (no primary antibody) are shown as indicated. Scale bars, 40 μm. Image adjustment in PowerPoint: all, brightness 50%. See Figure S4 for the staining of these markers in the iMG from the other donors. (C) Principal component analysis of vst-transformed RNA-seq read counts of the 2,000 most variable genes. iPSC (*n* = 9), iMG (*n* = 9) and MDMi (*n* = 4) samples from this study were integrated with samples from Vahsen et al. (Vahsen et al., 2022) (GSE200037; macrophage precursors: vahsen.pMacpre [*n* = 4], microglia in monoculture: vahsen.pMGl [*n* = 4], and co-culture with MNs: vahsen.copMG [*n* = 4]), Abud et al. (Abud et al., 2017) (GSE89189; microglia in monoculture: abud.iMGL [*n* = 6], microglia in co-culture with rat neurons: abud.rat.copMG [*n* = 6], CD14^+^ blood monocytes: abud.CD14M [*n* = 5], CD16^+^ blood monocytes: abud.CD16M [*n* = 4], fetal primary human microglia: abud.fMG [*n* = 3], adult primary human microglia: abud.aMG [*n* = 3]) and Muffat et al. (Muffat et al., 2016) (GSE85839; microglia in monoculture: muffat.pMGL [*n* = 6]). (D) Heatmap of relative vst-transformed RNA-seq read counts across the same samples as in c) for a set of ∼1,300 microglial genes identified by Galatro et al. (Galatro et al., 2017), drawn using the Heatmap function from the R-package ComplexHeatmap (v2.20.0).

**Figure 2:**
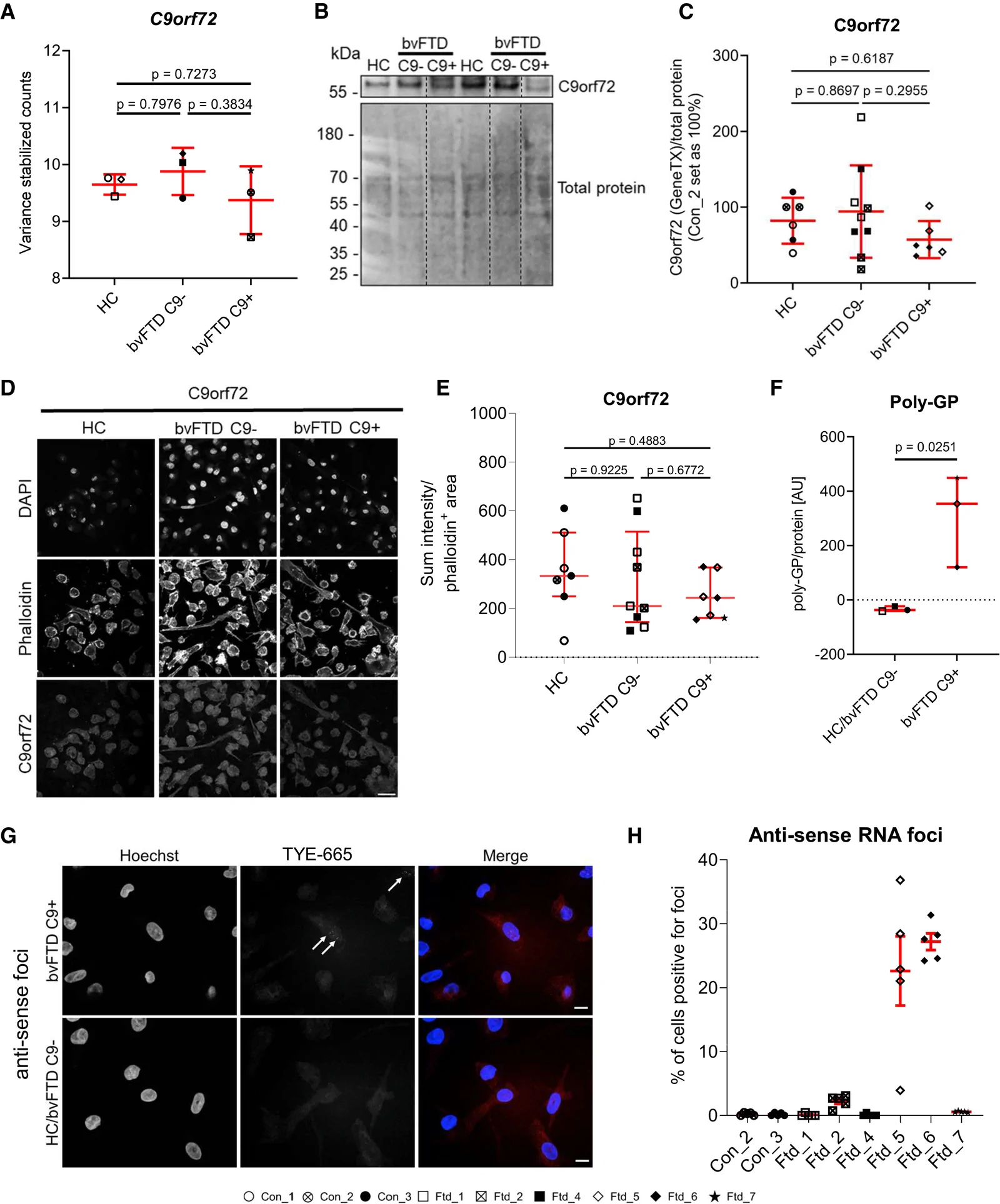
C9-HRE-related pathological hallmarks in iMG from healthy controls, sporadic (C9–) and C9-HRE (C9+) bvFTD patients (A) *C9orf72* gene expression for each donor according to RNA-seq shown as variance stabilized counts; *F* = 1.031, *p* = 0.4121; *n* (HC) = 3; *n* (bvFTD C9–) = 3; *n* (bvFTD C9+) = 3; each data point represents one RNA sample per donor. (B) Representative western blot image of C9orf72 protein levels in iMG. Dotted lines indicate cropping. Full western blot images are shown in Figures S5 and S6. (C) Quantification of western blot images from two to three independent differentiations. C9orf72 levels were normalized to total protein levels and to mean of normalized C9orf72 levels of samples from a healthy control; (*F* = 1.197, *p* = 0.3250; *n* [HC] = 6; *n* [bvFTD C9-] = 9; *n* [bvFTD C9+] = 6). (D) Representative IF images of C9orf72. Scale bars, 30 μm. Image adjustments in PowerPoint: all DAPI, brightness 40%, contrast 40%; all C9orf72, brightness 40%, contrast −40%. Phalloidin signal was used to normalize the quantified C9orf72 levels to cell confluency per region. (E) Quantification of C9orf72 levels based on IF images from three independent differentiations; (*H* = 0.7137, *p* = 0.5019; *n* [HC] = 7; *n* [bvFTD C9-] = 9; *n* [bvFTD C9+] = 7). (F) Poly-GP DPR proteins were detected in of C9-HRE iMG (bvFTD C9+). Measured poly-GP protein levels normalized to total protein concentration shown as arbitrary units from one healthy control (HC) iMG line (Con_3) and two sporadic bvFTD patient (bvFTD C9-) iMG lines (Ftd_1, Ftd_4) as C9-HRE non-carrying controls, and all three bvFTD C9+ iMG lines (Ftd_5, Ftd_6, Ftd_7) from one differentiation batch. Each data point represents the mean value of 3–4 measured samples per line. (G) Intranuclear anti-sense RNA foci (white arrows) in bvFTD C9+ iMG detected by fluorescence *in situ* hybridization using the TYE 665 probe. No signal was detected in bvFTD C9- iMG. Scale bars, 10 μm. (H) Quantification of anti-sense foci for each line. Each data point represents one measured sample. All data are from one differentiation batch. The individual iMG lines are indicated by different symbols at the bottom. For each group, descriptive statistics are shown as mean ± standard deviation (A, C, and E), median ± interquartile range (F) or mean ± standard error mean (H). One-way ANOVA followed by Tukey’s multiple comparisons test (A and C) or Kruskal-Wallis test followed by Dunn’s comparisons test (E) or two-tailed independent samples *t* test (F) were used for statistical analysis.

iMG generated (Konttinen et al., 2019) from iPSCs (Figures S1–S3) of three healthy donors, three bvFTD patients carrying the C9-HRE, and three sporadic bvFTD patients (Table S1) expressed myeloid lineage/microglial markers (Butovsky et al., 2014; Douvaras et al., 2017; Haenseler et al., 2017; Kerk et al., 2022; Konttinen et al., 2019; Masuda et al., 2020; Pandya et al., 2017) at the RNA (Figure 1A) and protein (Figures 1B and S4) level. Practically all cells (97%–100%) expressed IBA1 (Figure S4C). Global mRNA sequencing showed clustering of iMG close to blood monocyte-derived microglia-like cells and microglia from previously published datasets (Abud et al., 2017; Muffat et al., 2016; Vahsen et al., 2022) (Figures 1C and 1D). Typical C9-HRE-derived pathological hallmarks, i.e., sense and anti-sense RNA foci (DeJesus-Hernandez et al., 2011; Lagier-Tourenne et al., 2013; McEachin et al., 2020; Mizielinska et al., 2013), and DPR proteins (Gendron et al., 2013; Mori et al., 2013), but not *C9orf72* haploinsufficiency (DeJesus-Hernandez et al., 2011; Frick et al., 2018; Liu et al., 2020), were present in the iMG. Reduced *C9orf72* expression in C9-HRE iMG could not be detected at RNA (Figure 2A) nor at protein level (Figures 2B–2E; Figures S5–S7). Poly-GP proteins (Figure 2F) as well as anti-sense RNA foci, which were more prominently present than sense foci, were only detectable in C9-HRE iMG (Figures 2G and 2H; Figure S8). Approximately 25% of the C9-HRE iMG showed anti-sense and 6%–7% sense RNA foci.

### iMG of bvFTD patients do not display TDP-43 pathology

**Figure 3:**
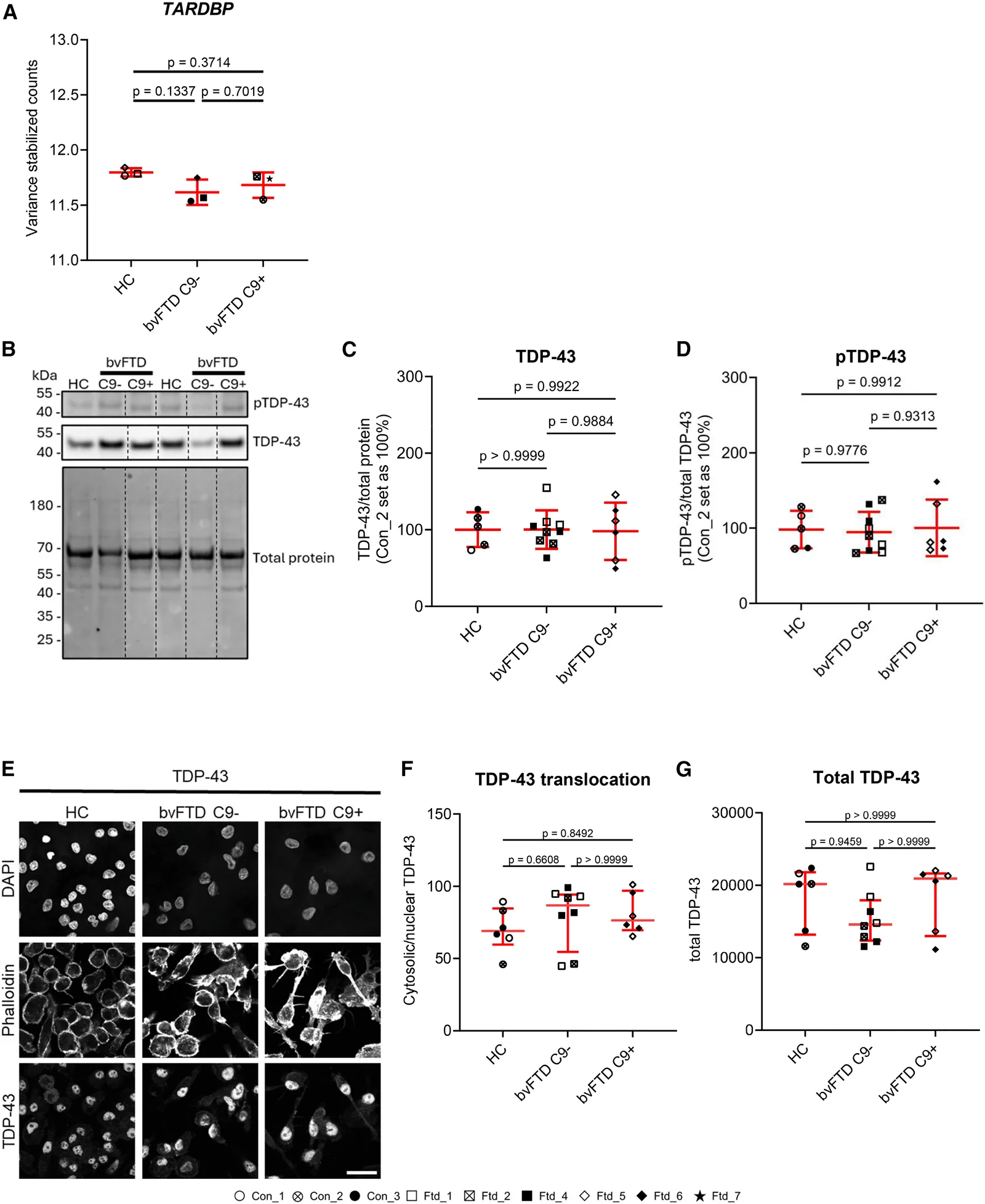
No TDP-43 pathology in iMG from healthy controls, sporadic (C9–) and C9-HRE (C9+) bvFTD patients (A) *TARDBP* gene expression according to RNA sequencing shown for each donor as mean ± standard deviation of variance stabilized counts; (*F* = 2.686, *p* = 0.1469; *n* [HC] = 3; *n* [bvFTD C9-] = 3; *n* [bvFTD C9+] = 3). (B) A representative western blot image to detect phosphorylated (Ser409/410-1; pTDP-43) and total TDP-43 levels in iMG. The blot(s) were re-probed for Figure 4B. Dotted lines indicate cropping. Full Western blot images are shown in Figures S9–S11, and S13. (C and D) Quantification of western blot images regarding total TDP-43 levels normalized to total protein levels (C) and pTDP-43 levels normalized to total TDP-43 levels (D). Each data point represents one biological replicate from one to three independent differentiation batches in total (C and D). Levels of total TDP-43/total protein (C) or pTDP-43/total TDP-43 (D) were normalized to mean levels of samples from a healthy control (Con_2). (For C: *F* = 0.01185, *p* = 0.9882. For D: *F* = 0.06744, *p* = 0.9350. For C, D: *n* [HC] = 5; *n* [bvFTD C9-] = 9; *n* [bvFTD C9+] = 6). (E) Representative microscopy images of TDP-43-stained iMG. DAPI and phalloidin signals were used to outline nuclei and cell boundaries. Scale bars, 30 μm. Image adjustments in PowerPoint: all TDP-43, brightness 30%. (F) Quantification of cytosolic-to-nuclear TDP-43 and (g) total TDP-43 based on IF imaging from three independent differentiations; (*n* [HC] = 6; *n* [bvFTD C9-] = 8; *n* [bvFTD C9+] = 6. For F: *H* = 1.748, *p* = 0.4347. For G: *H* = 1.342, *p* = 0.5331). The individual iMG lines are indicated by different symbols at the bottom. For each group, descriptive statistics are shown as mean ± standard deviation (A, C, and D) or median ± interquartile range (F and G). One-way ANOVA followed by Tukey’s multiple comparisons test (A, C, and D) or Kruskal-Wallis test followed by Dunn’s comparisons test (F and G) were used for statistical analysis.

Hyperphosphorylation, C-terminal fragmentation, and cytosolic aggregation of TDP-43 have been observed in neurons and glial cells in ALS and FTD patients (Berning and Walker, 2019; Cooper-Knock et al., 2012; Neumann et al., 2009, 2020). *TARDBP* gene expression was similar between all groups (Figure 3A). Total levels of full-length TDP-43 proteins were unchanged in all iMG (Figures 3B, 3C, and 3G; Figures S9 and S10) and did not show increased phosphorylation at serine 409/410-1 (Figures 3B and 3D; Figure S11) nor cleavage into smaller C-terminal fragments (<43 kDa) (Figure S9). No increased cytosolic-to-nuclear TDP-43 ratio was detected between bvFTD and control iMG on bulk (Figures 3E and 3F; Figure S7) or single cell level (Figure S12). These results collectively indicate a lack of TDP-43 pathology in these bvFTD iMG.

### iMG of bvFTD patients show changes in autophagosomal-lysosomal pathways

**Figure 4:**
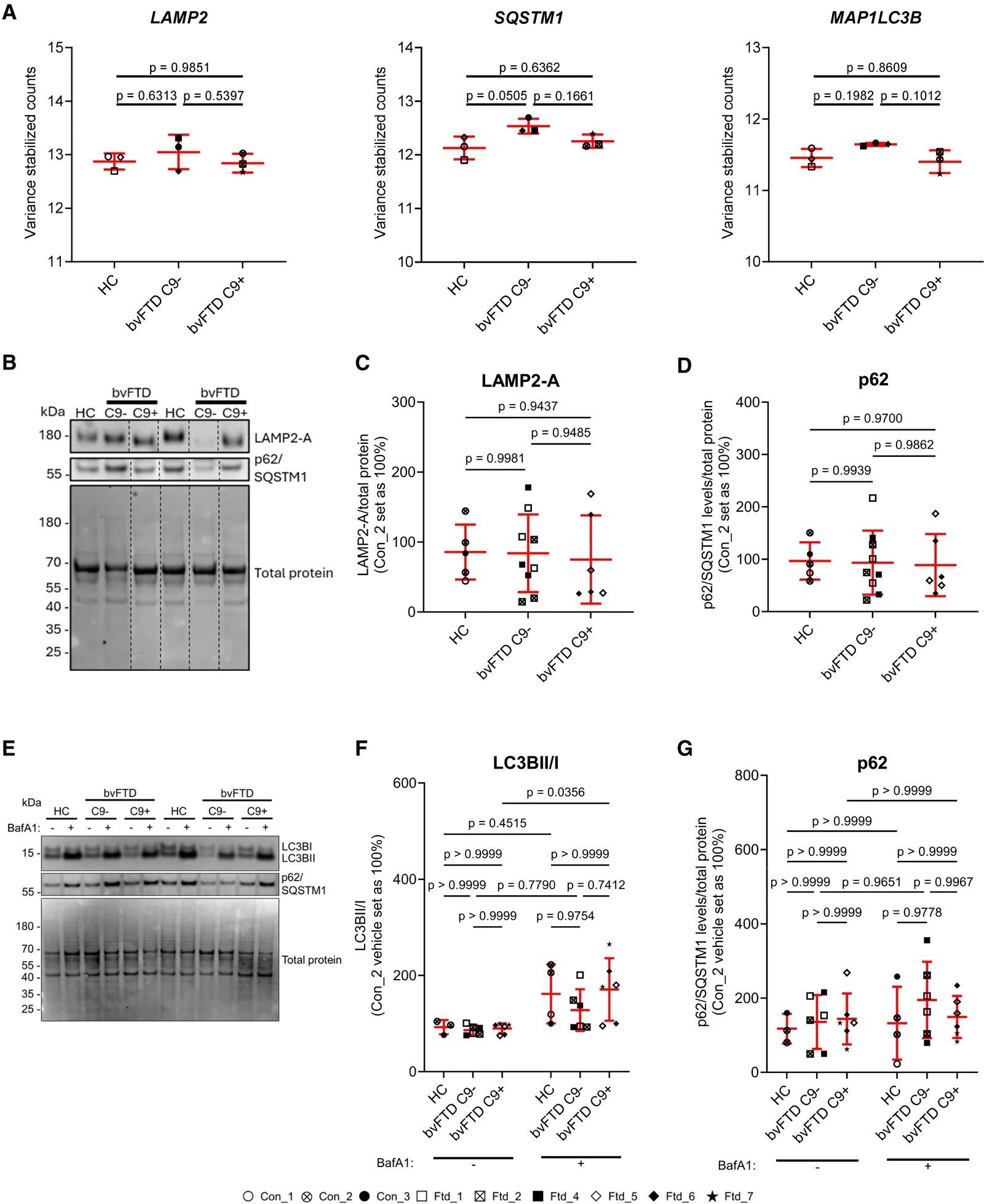
iMG from healthy controls, sporadic (C9–) and C9-HRE (C9+) bvFTD patients show normal autophagy (A) *LAMP2*, *SQSTM1*, and *MAP1LC3B* gene expression according to RNA-seq shown for each donor as variance stabilized counts; *LAMP2*: *F* = 0.7255, *p* = 0.5222; *SQSTM1*: *F* = 4.914, *p* = 0.0545; *MAP1LC3B*: *F* = 3.493, *p* = 0.0986; *n* (HC) = 3; *n* (bvFTD C9-) = 3; *n* (bvFTD C9+) = 3; each data point represents one RNA sample per donor. (B) Representative western blot images of LAMP2-A, p62/SQSTM1, and total protein signal. The blot(s) were re-probed for Figure 3B. Dotted lines indicate cropping. Full western blot images are shown in Figures S10, S13, and S14. (C and D) Quantification of LAMP2-A (C) and p62/SQSTM1 (D) signals normalized to total protein levels. (E) Representative western blot images of LC3BI and II ± bafilomycin A1 (BafA1) treatment. (F) Quantification of LC3BII/I ratio after normalization to total protein levels. Each data point represents one biological replicate from one to three (two for E, F, and G) independent differentiation batches. (For C: *F* = 0.06633, *p* = 0.9361; D: *F* = 0.02856, *p* = 0.9719; F: *F* [treatment] = 17.00, *p* = 0.0004, *F* [group] = 1.042, *p* = 0.3675, *F* [interaction] = 0.6988, *p* = 0.5066; G: *F* [treatment] = 0.8177, *p* = 0.3745, *F* [group] = 0.5978, *p* = 0.5577, *F* [interaction] = 0.3960, *p* = 0.6772. For C, D: *n* [HC] = 5; *n* [bvFTD C9-] = 9; *n* [bvFTD C9+] = 6. For F, G: *n* [HC, vehicle] = 3; *n* [HC, BafA1] = 4; *n* [bvFTD C9-, vehicle] = 6; *n* [bvFTD C9-, BafA1] = 6; *n* [bvFTD C9+, vehicle] = 6; *n* [bvFTD C9+, BafA1] = 6). The individual iMG lines are indicated by different symbols at the bottom. For each group, descriptive statistics are shown as mean ± standard deviation (A, C, D, F, and G). One-way ANOVA followed by Tukey’s multiple comparisons test (A, C, and D) or two-way ANOVA followed by Sidak’s multiple comparisons test (F and G) were used for statistical analysis.

**Figure 5:**
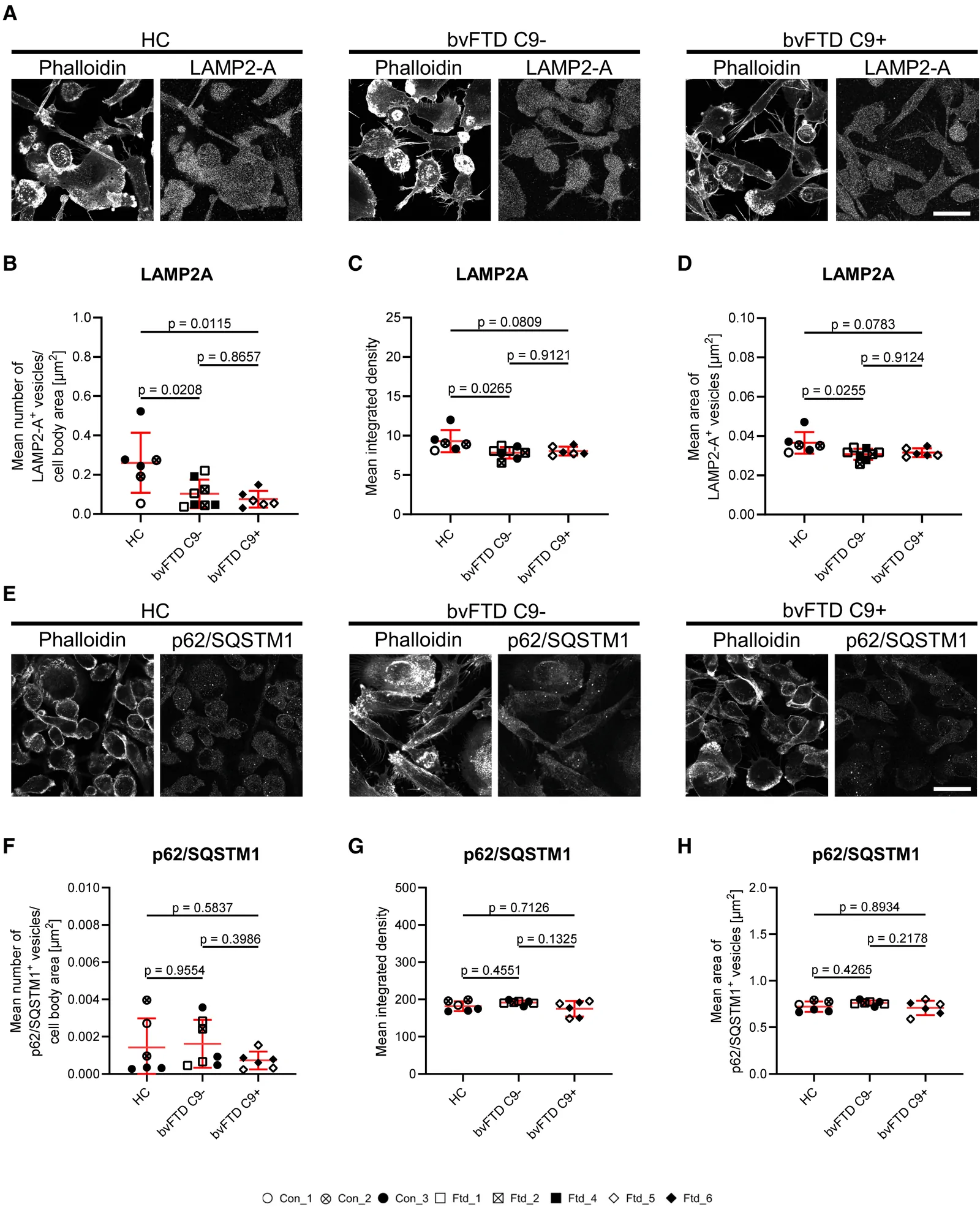
Lysosomes in iMG of healthy controls, sporadic (C9–) and C9-HRE (C9+) bvFTD patients (A–E) Representative IF images are shown per group for (A) LAMP2-A and (E) p62/SQSTM1. Phalloidin staining was used to outline cell boundaries. Scale bars, 30 μm. Image adjustments in PowerPoint: all LAMP2-A, brightness 80%; all p62/SQSTM1, brightness 50%. Data from three independent differentiations. (For B: *F* = 6.488, *p* < 0.0081; C: *F* = 4.575, *p* < 0.0257; D: *F* = 4.636, *p* < 0.0247. For B–D: *n* [HC] = 6; *n* [bvFTD C9-] = 8; *n* [bvFTD C9+] = 6. For F: *F* = 0.9552, *p* = 0.4056, . For G: *F* = 2.151, *p* = 0.1488,. For H: *F* = 1.673, *p* = 0.2189. For F–H: *n* [HC] = 6; *n* [bvFTD C9-] = 7; *n* [bvFTD C9+] = 6). The individual iMG lines are indicated by different symbols at the bottom. For each group, descriptive statistics are shown as mean ± standard deviation. One-way ANOVA followed by Sidak’s multiple comparisons test was used for statistical analysis.

All iMG showed similar expression of *SQSTM1*, *MAP1LC3B*, and *LAMP2* RNA (Figure 4A) and proteins (Figures 4B–4D; Figures S10, S13, and S14). No difference in LC3BI to II conversion, which would indicate alterations in the autophagosomal activity, was detected between bvFTD and control iMG under basal conditions. However, C9-HRE iMG showed a significant increase in LC3BII to I conversion after Bafilomycin A1 treatment (Figures 4E and 4F). We found no differences in protein levels of p62/SQSTM1 after Bafilomycin A1 treatment between the disease groups (Figures 4E and 4G). Quantitative IF analysis showed reduced number of LAMP2-A-positive vesicles in bvFTD iMG compared to iMG of healthy controls, and smaller vesicles with less LAMP2-A positivity in iMG from sporadic bvFTD patients (Figures 5A–5D; Figure S7). No differences between the groups were detected regarding p62 vesicles (Figures 5E–5H; Figure S7). These data altogether indicate that iMG of bvFTD patients might exhibit changes in autophagosomal-lysosomal pathways.

### iMG of bvFTD patients show altered phagocytosis

**Figure 6:**
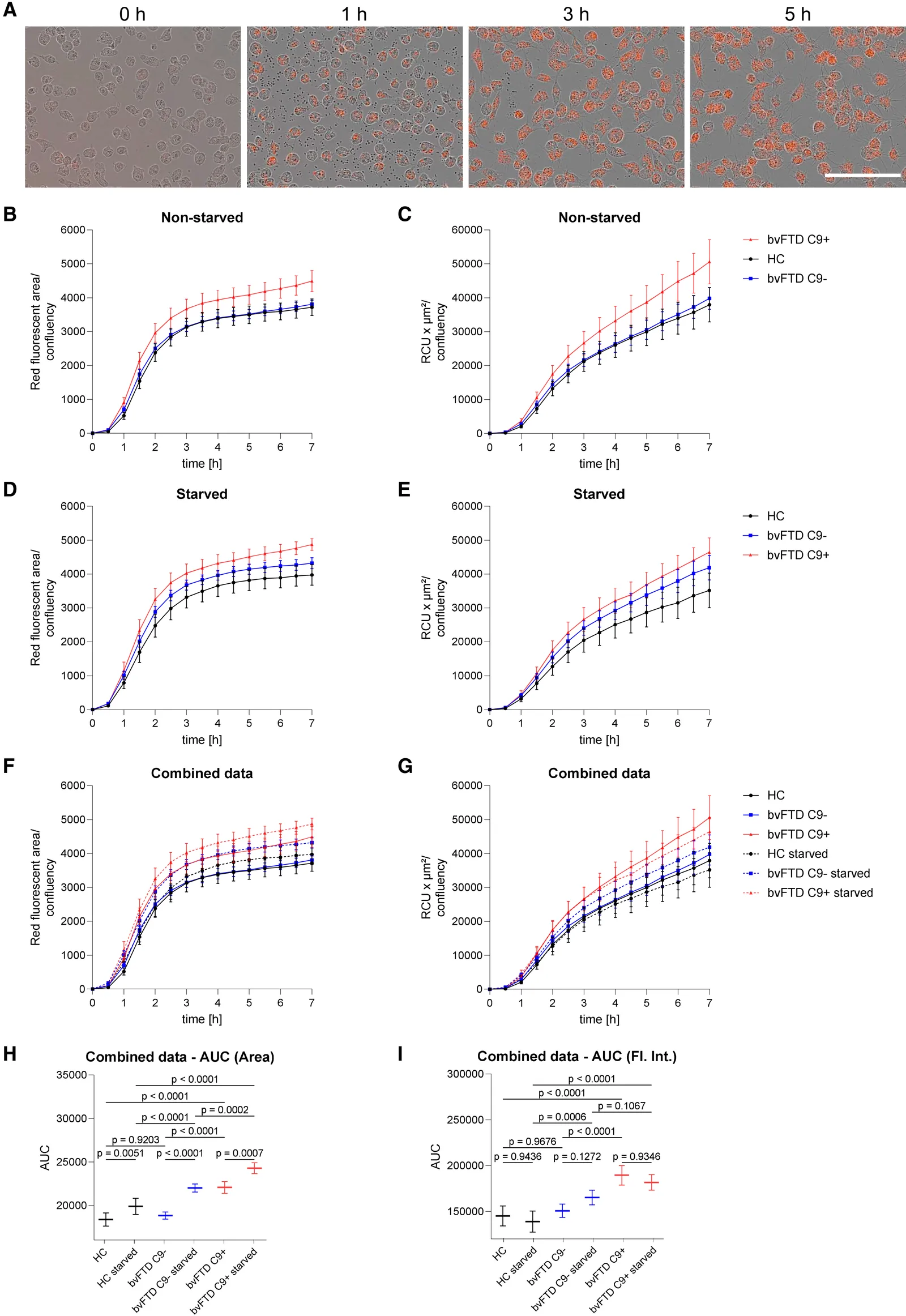
Phagocytosis in iMG of healthy controls, sporadic (C9–) and C9-HRE (C9+) bvFTD patients iMG cultured under normal conditions were incubated with pHrodo zymosan beads and imaged over time. (A–I) Representative images of iMG from a healthy donor. Scale bars, 150 μm. For each well, red fluorescence was measured over time and normalized to confluency prior to zymosan bead addition. Area of red fluorescence (B, D, F, and H) or fluorescence intensity (C, E, G, and I). Data are shown as mean ± SEM or standard deviation (H and I). Statistical testing (one-way ANOVA followed by Sidak’s multiple comparisons test) after area under the curve analysis (H: *F* = 55.80, *p* < 0.0001, I: *F* = 21.83, *p* < 0.0001). Data are shown from two independent differentiation batches (*n* [HC] = 6; *n* [bvFTD C9-] = 6; *n* [bvFTD C9+] = 4).

Several studies have suggested involvement of C9orf72 in phagocytosis (Laflamme et al., 2019; Lall et al., 2021). Hence, phagocytic activity in the bvFTD iMG was characterized by following the uptake of pH-sensitive zymosan-coupled bioparticles over time. All iMG first showed increased fluorescence, indicating phagocytic uptake of the particles and their fusion with lysosomes, and subsequently increased number and length of motile processes (Figure 6A; Videos S1, S2, S3, S4, S5, S6, S7, S8, S9, S10, S11, S12, S13, S14, S15, and S16). C9-HRE iMG showed an increased area (Figures 6B and 6H) and intensity (Figures 6C and 6I) of the fluorescence signal when compared to iMG from healthy controls and sporadic bvFTD patients. We used serum starvation to enhance potential autophagolysosomal differences in bvFTD iMG and *C9orf72* loss-of-function-related changes, as we have previously shown altered serum starvation-induced autophagy in neuronal cells with decreased C9orf72 expression (Leskelä et al., 2019). While serum starvation was previously shown to dampen phagocytosis of human iPSC-derived microglia *in vitro* (Brownjohn et al., 2018), we observed an increased fluorescent area in all iMG after serum starvation compared to the non-starved cells (Figures 6D, 6F, and 6H). Moreover, sporadic bvFTD iMG also showed an increased area (Figures 6D, 6F, and 6H) and intensity (Figures 6E, 6G, and 6I) of the fluorescent signal as compared to healthy controls under serum starvation. C9-HRE iMG showed the highest fluorescent signal compared to the other iMG also after serum starvation. These results suggest that iMG of bvFTD patients show altered phagocytosis under basal and autophagy-inducing conditions.

Video S1. Phagocytosis assay of Con_1 line without prior serum starvation

Video S2. Phagocytosis assay of Con_1 line after 24 h serum starvation

Video S3. Phagocytosis assay of Con_2 line without prior serum starvation

Video S4. Phagocytosis assay of Con_2 line after 24 h serum starvation

Video S5. Phagocytosis assay of Con_3 line without prior serum starvation

Video S6. Phagocytosis assay of Con_3 line after 24 h serum starvation

Video S7. Phagocytosis assay of Ftd_1 line without prior serum starvation

Video S8. Phagocytosis assay of Ftd_1 line after 24 h serum starvation

Video S9. Phagocytosis assay of Ftd_2 line without prior serum starvation

Video S10. Phagocytosis assay of Ftd_2 line after 24 h serum starvation

Video S11. Phagocytosis assay of Ftd_4 line without prior serum starvation

Video S12. Phagocytosis assay of Ftd_4 line after 24 h serum starvation

Video S13. Phagocytosis assay of Ftd_5 line without prior serum starvation

Video S14. Phagocytosis assay of Ftd_5 line after 24 h serum starvation

Video S15. Phagocytosis assay of Ftd_6 line without prior serum starvation

Video S16. Phagocytosis assay of Ftd_6 line after 24 h serum starvation

### C9-HRE iMG exhibit changed gene expression related to phagocytosis and autophagy

Next, we investigated whether genes related to phagocytosis and autophagy were differentially expressed in the bvFTD iMG. No differences in the expression of zymosan-binding receptors (Brown et al., 2002; Ozinsky et al., 2000) could be detected (Figure S15A). Pathway analysis of genes involved in phagocytosis regulation and recognition and autophagy showed that cluster of differentiation (*CD*) *93*, ras-related C3 botulinum toxin substrate 2 (*RAC2*), *CD300A*, coronin 1C (*CORO1C*), heat shock protein 90 alpha family class A member 1 (*HSP90AA1*), and tubulin alpha 1b (*TUBA1B*) were significantly upregulated in C9-HRE iMG as compared to iMG from sporadic bvFTD patients but not healthy control iMG (Figure S15B; Table S2).

### bvFTD iMG show differential gene expression related to RNA and protein regulation and cellular energy metabolism

**Figure 7:**
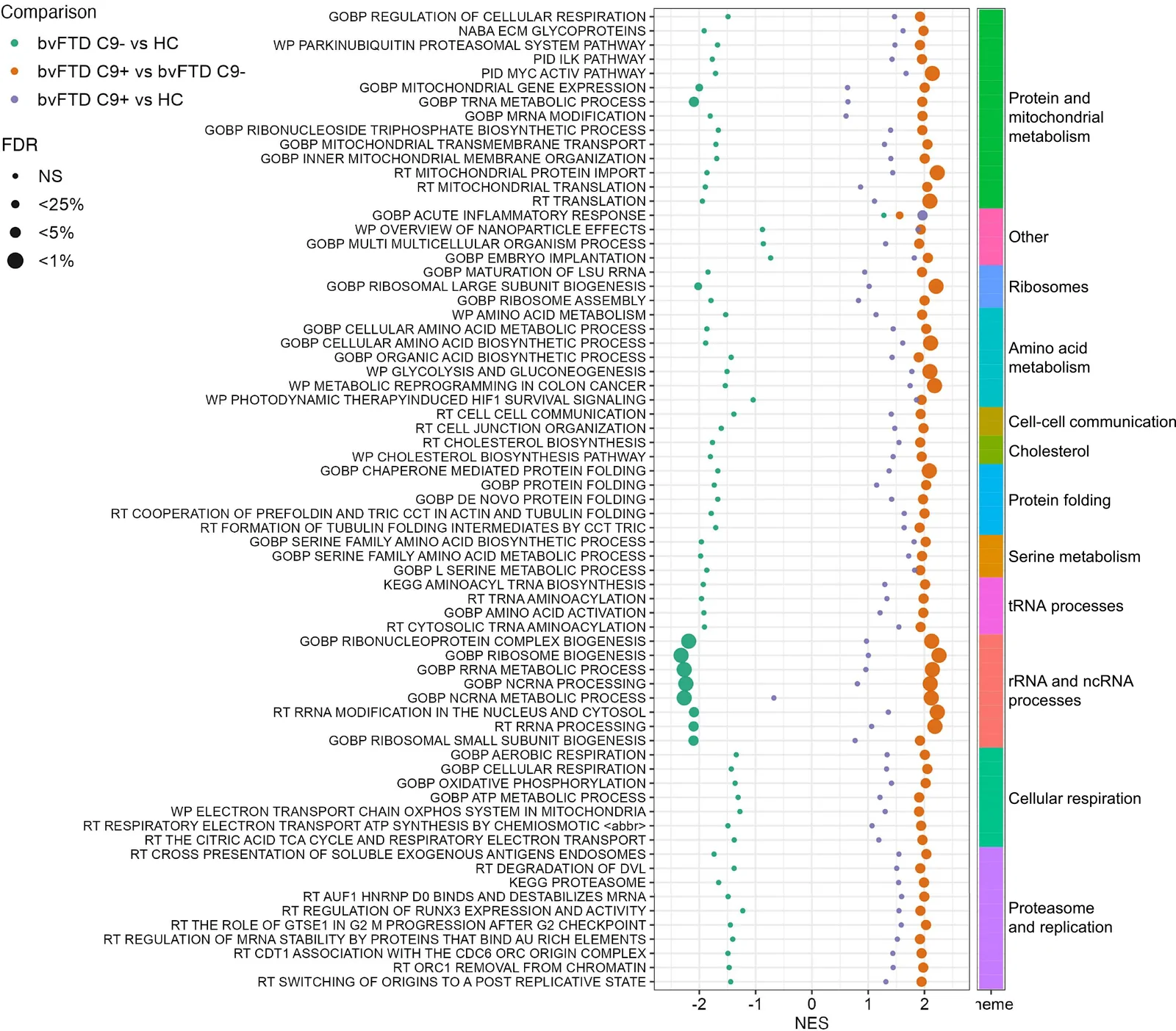
Differentially enriched pathways in iMG of healthy controls, sporadic (C9–), and C9-HRE- (C9+) bvFTD patients The dot plot displays, for the three differently colored comparisons, the gene set enrichment analysis (GSEA) results for the MSigDB gene set collections GO biological processes (C5 GO BP) and canonical pathways (C2 CP). Normalized enrichment score is shown in the *x* axis whereas the categorical statistical significance (FDR) is indicated by the size of the marker. Data are shown from one differentiation batch.

Gene set enrichment analysis revealed that pathways involved in the regulation of different RNA (tRNA, rRNA, mRNA, and ncRNA) processes and protein translation, localization, and degradation as well as genes involved in energy metabolism, including mitochondrial metabolism, were specifically upregulated in C9-HRE iMG compared to sporadic bvFTD iMG. Interestingly, the iMG of sporadic bvFTD patients showed a decreased expression pattern in pathways related to rRNA, tRNA, and ncRNA metabolism compared to iMG of healthy controls (Figure 7).

### C9-HRE iMG display differential expression of genes involved in acute inflammatory response

In comparison to healthy controls, C9-HRE iMG showed a higher expression of genes involved in acute inflammatory response, namely orosomucoid 1 (*ORM1*), prostaglandin-endoperoxide synthase 2 (*PTGS2*), and S100 calcium-binding protein A8 (*S100A8*) (Figure 7; Figure S16A; Table S2). However, the RNA levels of cytokines and chemoattractants related to microglial activation and previously found altered in C9-HRE carriers (Gray et al., 2020; Huang et al., 2020; Ismail et al., 2013; Olesen et al., 2020) were not altered in bvFTD patient iMG compared to control iMG. Only *IL1B* showed higher levels in the C9-HRE iMG as compared to sporadic bvFTD patients but not as compared to healthy controls (Figure S16B; Table S2). Also, no obvious changes in the inflammation phenotype-related interferon response microglia or active response microglia signatures (Lall et al., 2021; McCauley et al., 2020) could be detected in C9-HRE iMG compared to the iMG from sporadic bvFTD patients or healthy controls (Table S2).

## Discussion

In this study, we generated for the first time iMG from sporadic and C9-HRE-carrying bvFTD patients without concomitant ALS diagnosis. We confirmed that all iMG similarly expressed typical microglial genes and that iMG from C9-HRE carriers exhibited typical C9-HRE-derived pathological hallmarks, including RNA foci and DPRs, but no reduced C9orf72 levels, similar to previous studies (Lorenzini et al., 2023; Vahsen et al., 2023). No evident signs of TDP-43 pathology were detected in the bvFTD iMG in accordance with previous reports on C9-HRE iMG (Lorenzini et al., 2023; Vahsen et al., 2023). Whereas no difference in LC3BII/I conversion was detected in bvFTD iMG vs. control iMG under basal conditions, only C9-HRE iMG showed significantly increased LC3BII/I conversion after Bafilomycin A1 treatment. IF revealed a reduced number LAMP-2A^+^ vesicles in bvFTD iMG, similar to findings in ALS iPSC-derived motoneurons (Shi et al., 2018, 2019). Functionally, we found that bvFTD iMG showed differences in phagocytosis under serum starvation and in the C9-HRE iMG already under basal conditions. The increased fluorescence signal could be an indication of higher acidification of the phagolysosomes as the beads used are pH-sensitive, or reduced lysosomal degradation as shown in *C9orf72* knockout iMG (Masrori et al., 2025). Previously, reduced fluorescence upon phagocytosis of zymosan beads was detected with 10-fold higher concentrations of the beads as compared to our study and this reduction was observed already after 30 to 75 min. In the same study, C9-HRE iMG showed reduced LC3BII/I conversion after Bafilomycin A1 treatment. These differences might be explained by the prominently lower C9orf72 levels or the absence of FBS in the differentiation protocol in that study (Banerjee et al., 2023). Some differentially expressed genes and pathways related to phagocytosis and autophagy in the iMG from C9-HRE bvFTD patients compared to iMG sporadic bvFTD patients were detected, which could explain the observed altered autophagy and phagocytic activity in the C9-HRE iMG. Future proteomic approaches might be useful to further investigate the underlying mechanisms related to these findings.

According to gene set enrichment analysis, C9-HRE iMG showed remarkable differences compared to sporadic bvFTD iMG related to RNA and protein regulation and energy metabolism. Recent single cell RNA sequencing of iMG with C9-HRE ALS background compared to isogenic controls also revealed differences in protein regulation and metabolism (Mearelli et al., 2025). However, similarly to a previous report, only a few genes were differentially expressed compared to healthy control iMG (Lorenzini et al., 2023). In contrast, sporadic bvFTD iMG showed marked differences vs. healthy control iMG, suggesting an opposite RNA profile to the C9-HRE iMG. Although some alterations in genes related to acute inflammatory response in C9-HRE iMG vs. healthy control iMG were detected, no overt inflammatory phenotype in bvFTD iMG was observed. Together, these findings suggest alterations in lysosomal degradation, phagocytosis, and metabolism in bvFTD patient-derived iMG. The differences between iMG from sporadic vs. C9-HRE patients might derive from their different genetic backgrounds potentially involving different molecular-level alterations, warranting further characterization in future studies.

One limitation of our study is the use of iMG monocultures. Previous studies have indicated that microglia readily respond to their environment and that microglia *in vitro* show different RNA expression profiles than brain-resident microglia *in vivo* (Timmerman et al., 2018). Thus, a neuron-astrocyte-microglia co-culture approach might give deeper insights into disease pathogenesis and underlying mechanisms. Since co-culturing C9-HRE ALS iMG with motoneurons increased neuronal vulnerability (Banerjee et al., 2023; Mearelli et al., 2025; Vahsen et al., 2023), it would be important to also examine co-cultures from bvFTD patients. On the other hand, we observed that the iMG of bvFTD patients in monocultures showed differences in potential disease-relevant pathways, such as phagocytosis and RNA, protein, and energy metabolism, as compared to controls. This suggests that despite representing a likely immature and simplified model system, monocultures of iMG may provide important initial insights into potential microglial alterations in bvFTD that may be further examined in more complex models and human brain samples to evaluate the translational value of the findings. Also, isogenic lines of C9-HRE iPSCs could be used as additional controls in addition to the healthy control lines. This might enable identification of pathways specifically altered due to the C9-HRE. Targeting the formation of RNA foci and DPR proteins through e.g., antisense oligonucleotides might also shed light onto underlying molecular mechanisms of the observed phenotypes in C9-HRE iMG. Furthermore, exposing C9-HRE iMG to different inflammatory stimuli might provide insights into possible differential activation states or function compared to the iMG from sporadic bvFTD patients or healthy controls. Finally, since high variance was detected in some experiments, inclusion of a larger number of iMG lines per group might increase the statistical power in future experiments.

Taken together, we report here for the first time comparison of the RNA profiles and cell pathological and functional phenotypes of the iMG derived from different bvFTD patients. The present study represents a starting point for using patient-derived iMG as a disease model for bvFTD with different genetic backgrounds. Future investigations will advance understanding of the underlying molecular mechanisms and the involvement of microglia in the pathogenesis of different clinical and genetic forms of bvFTD.

## Resource availability

### Lead contact

Requests for further information and resources should be directed to and will be fulfilled by the lead contact, Annakaisa Haapasalo (annakaisa.haapasalo@uef.fi).

### Materials availability

This study did not generate new unique reagents.

### Data and code availability

Requests for further information and resources should be directed to and will be fulfilled by the lead contact. All data reported in this study will be shared by the lead contact upon reasonable request. RNA sequencing (RNA-seq) data are available at https://ega-archive.org/search/EGAC50000000949.

## Acknowledgments

We would like to thank all the study participants for their valuable blood and skin biopsy donations. Thank you to Laila Kaskela and Eila Korhonen for iPSC generation and Jenni Voutilainen and Ida Hyötyläinen for assistance characterizing the generated iPSC lines. H.R., S.L., N.H., and S.O. were supported by the 10.13039/100007753University of Eastern Finland (UEF) Doctoral Programme in Molecular Medicine (DPMM). H.R. was supported by the GenomMed doctoral program. UEF Cell and Tissue Imaging Unit, supported by Biocenter Kuopio and Biocenter Finland is acknowledged for providing IncuCyte S3 and LSM800 training and facilities. UEF Bioinformatics Center is acknowledged for the use of the high-performance computing cluster. This publication is part of a project that has received funding from the 10.13039/501100007601European Union’s Horizon 2020 research and innovation program under the Marie Skłodowska-Curie grant agreement no 740264. This study is part of the research activities of the Finnish FTD Research Network (FinFTD).

The EBiSC Bank acknowledges University College London as the source of the human induced pluripotent cell line UCLi001-A, which was generated at UCL Queen Square Institute of Neurology with support from the NIHR 10.13039/501100012317UCLH Biomedical Research Centre, 10.13039/100013322EFPIA companies and the European Union (IMI-JU). This study was supported by academic research grants from the 10.13039/501100002341Research Council of Finland, grant nos. 315459 (A.H.), 315460 (A.M.P.), 288659 (T.N.), 328287 (T.M.), 330178 (M.T.) and 338182 (M.H.); 10.13039/100010114Yrjö Jahnsson Foundation, grant no. 20187070 (A.H.); 10.13039/501100004212Päivikki and Sakari Sohlberg Foundation (A.H.); 10.13039/501100004012Jane and Aatos Erkko Foundation (A.H.); ALS tutkimuksen tuki ry. registered association (H.R., S.L., and N.H.); The 10.13039/100010129Maud Kuistila Memorial Foundation (H.R.); 10.13039/501100007083Orion Research Foundation sr (H.R. and E.S.); The 10.13039/501100003125Finnish Cultural Foundation (T.H.); Kuopio University Foundation (H.R.); 10.13039/501100006306Sigrid Jusélius Foundation (A.H., M.H., E.S., and Š.L.); The Finnish Brain Foundation (E.S. and K.K.); 10.13039/501100008413Instrumentarium Science Foundation (E.S.); The 10.13039/100008723Finnish Medical Foundation (E.S. and K.K.); Maire Taponen Foundation (K.K.); The Strategic Neuroscience Funding of the 10.13039/100007753University of Eastern Finland (A.H. and M.H.); and Neurocenter Finland – AlzTrans pilot project (M.H.).

## Author contributions

Conceptualization, A.H. and H.R.; methodology, H.R., H.J., S.O., S.L., N.H., Š.L., J.K., T.M., P.H., H.M., M.T., N.H., A.D., S.R.-N., P.M., and M.H.; formal analysis, H.R., T.H., T.K., and S.H.; investigation, H.R., T.H., N.H., D.H., A.D., S.R.-N., Š.L., V.P., P.M., E.S., and K.K.; resources, A.H., M.H., A.M.P., P.H., Š.L., and J.K.; data curation, A.H. and S.H.; writing – original draft, H.R.; writing – review and editing, H.R., T.H., A.M.P., E.S., K.K., S.H., and A.H.; visualization, H.R., T.H., S.H., and Š.L.; funding acquisition, A.H., A.M.P., and M.H.; supervision, Š.L., T.N., H.M., M.T., T.M., A.H., and M.H.

## Declaration of interests

The authors declare no competing interests.

## STAR★Methods

### Key resources table

**Table:** 

REAGENT or RESOURCE	SOURCE	IDENTIFIER
**Antibodies**		

Monoclonal, mouse, anti-C9orf72	GeneTex	Cat# GTX634482 RRID: AB_2784545
Polyclonal, rabbit, anti-LAMP2-A	Abcam	Cat# ab18528 RRID: AB_775981
Polyclonal, rabbit, anti-LC3B	Abcam	Cat# ab51520 RRID: AB_881429
Polyclonal, rabbit, anti-phospho-TDP-43	CosmoBio	Cat# TIP-PTD-P02 RRID: AB_1961898
Polyclonal, rabbit, anti-SQSTM1	CST	Cat# 5114S RRID: AB_10624872
Polyclonal, rabbit, anti-TDP-43	Proteintech	Cat# 10782-2-AP RRID: AB_615042
Goat Anti-Rat IgG, HRP Conjugated	GE Healthcare	Cat# NA935 RRID: AB_772207
Donkey Anti-Rabbit IgG, HRP Conjugated	GE Healthcare	Cat# NA934V RRID: AB_772206
Sheep Anti-Mouse IgG, HRP Conjugated	GE Healthcare	Cat# NA931V RRID: AB_772210
Monoclonal, rabbit, anti-CX3CR1	Thermo Fisher	Cat# 702321 RRID: AB_2662897
Monoclonal, mouse, anti-IBA1	Thermo Fisher	Cat# MA5-27726 RRID: AB_2735228
Polyclonal, goat, anti-NANOG	R&D Systems	Cat# AF1997 RRID: AB_355097
Polyclonal, rabbit, anti-P2RY12	Sigma-Aldrich	Cat# HPA014518 RRID: AB_2669027
Monoclonal, mouse, anti*-*POU5F1	EMD Millipore	Cat# MAB4401 RRID: AB_2167852
Monoclonal, mouse, anti-SSEA4	EMD Millipore	Cat# MAB4304 RRID: AB_177629
Monoclonal, mouse, anti-SQSTM1	Santa Cruz Biotechnology	Cat# sc-28359 RRID: AB_628279
Polyclonal, rabbit, anti-TMEM119	Thermo Fisher	Cat# HPA051870 RRID: AB_2681645
Monoclonal, mouse, anti-TRA-1-81	EMD Millipore	Cat# MAB4381 RRID: AB_177638
Polyclonal, goat, anti-TREM2	Thermo Fisher	Cat# PA5-46980 RRID: AB_2576895
Goat anti-Mouse IgG (H + L) Cross-Adsorbed Secondary Antibody, Alexa Fluor™ 488	Invitrogen	Cat# A-11001 RRID: AB_2534069
Goat anti-Rat IgG (H + L) Cross-Adsorbed Secondary Antibody, Alexa Fluor™ 488	Invitrogen	Cat# A-11006 RRID: AB_2534074
Goat anti-Rabbit IgG (H + L) Highly Cross-Adsorbed Secondary Antibody, Alexa Fluor™ 488	Invitrogen	Cat# A-11034 RRID: AB_2576217
Donkey anti-Goat IgG (H + L) Cross-Adsorbed Secondary Antibody, Alexa Fluor™ 488	Invitrogen	Cat# A-11055 RRID: AB_2534102
Rabbit anti-Mouse IgG (H + L) Cross-Adsorbed Secondary Antibody, Alexa Fluor™ 488	Invitrogen	Cat# A-11059 RRID: AB_2534106
Goat anti-Mouse IgG (H + L) Cross-Adsorbed Secondary Antibody, Alexa Fluor™ 568	Invitrogen	Cat# A-11004 RRID: AB_2534072
Donkey anti-Goat IgG (H + L) Cross-Adsorbed Secondary Antibody, Alexa Fluor™ 568	Invitrogen	Cat# A-11057 RRID: AB_2534104
Goat anti-Rabbit IgG (H + L) Cross-Adsorbed Secondary Antibody, Alexa Fluor™ 647	Invitrogen	Cat# A-21244 RRID: AB_2535812

**Chemicals, peptides, and recombinant proteins**		

Matrigel	Corning	Cat# 356230
E8 medium with E8 supplement	Gibco	Cat# A1517001
Penicillin/streptomycin	Gibco	Cat# 15140122
EDTA	Invitrogen	Cat# 15575020
DPBS	Lonza	Cat# 17-512F
Y-27632 2HCl	Selleck Chemicals	Cat# S1049
Heat inactivated FBS	Gibco	Cat# 10500
DMSO	Sigma	Cat# D2650
Vybrant DyeCycle Green Stain	Thermo Fisher	Cat# V35004
Activin A	Peprotech	Cat# 120-14E
BMP4	Peprotech	Cat# 120-05ET
CHIR99021	Axon	Cat# 1386 B8
DMEM F-12	Gibco	Cat# 21331-020
GlutaMAX	Life Technologies	Cat# 35050-038
Ascorbic acid	Sigma-Aldrich	Cat# A8960
Sodium selenite	Sigma-Aldrich	Cat# S5261
Sodium bicarbonate	Thermo Fisher	Cat# 25080-094
FGF2	Peprotech	Cat# AF-100-18B
SB431542	Selleckchem	Cat# S1067
Insulin	Sigma-Aldrich	Cat# I9278
VEGF	Peprotech	Cat# 100-20A
TPO	Peprotech	Cat# 300-18
SCF	Peprotech	Cat# 300-07
IL-6	Peprotech	Cat# 200-06
IL-3	Peprotech	Cat# 200-03
IMDM	Gibco	Cat# 21980032
MCSF	Peprotech	Cat# 300-25
IL-34	Peprotech	Cat# 200-34
PDL	Sigma	Cat# P6407
Bafilomycin A1	Sigma Aldrich	Cat# B1793-10UG
ECL™ Prime Western Blotting Detection Reagent	GE Healthcare	Cat# RPN2236
ECL™ Select Western Blotting Detection Reagent	GE Healthcare	Cat# RPN2235
UltraScence Pico Ultra	BioHelix	Cat# CCH345
No-Stain Protein Labeling Reagent	Invitrogen	Cat# A44449
Formaldehyde	Thermo Scientific	Cat# 28908
Diethyl pyrocarbonate	Sigma	Cat# D5758
Goat immunoglobulin G isotype control	Invitrogen	Cat# 02-6202
Mounting medium	Vector Laboratories	Cat# H-1800
DAPI	Sigma	Cat# D952
Phalloidin-containing mounting medium	Vector Laboratories	Cat# H-1600

**Critical commercial assays**		

AmplideX PCR/CE C9orf72 Kit	Asuragen	Cat# 49581
CytoTune-iPS Sendai reprogramming kit	Thermo Fisher	Cat# A16517
Zymosan-coupled pH-sensitive fluorescent particles	Invitrogen	Cat# P35364
NucleoProtect RNA	MACHEREY-NAGEL	Cat# 740400.250
RNA extraction kit	Roche Molecular Systems, Inc.	Cat# 11828665001

**Deposited data**		

RNA seq data from Vahsen et al.	(Vahsen et al., 2022)	GSE200037
RNA seq data from Abud et al.	(Abud et al., 2017)	GSE89189
RNA seq data from Muffat et al.	(Muffat et al., 2016)	GSE85839
RNA seq data from this paper	This paper	https://ega-archive.org/search/EGAC50000000949
Gencode human transcriptome version 38 (for genome version hg38)	(Frankish et al., 2019)	https://www.gencodegenes.org/
Molecular Signatures Database gene sets (MSigDB, version 7.5.1)	(Liberzon et al., 2011; 2015; Subramanian et al., 2005)	https://www.gsea-msigdb.org/

**Experimental models: Cell lines**		

UCLi001-A, Biosamples ID: SAMEA3174431	EBiSC	https://ebisc.org/

**Oligonucleotides**		

TYE 563-(CCCCGG)_3_	Exiqon	N/A
TYE 665-(CCCCGG)_2.5_	Qiagen	Cat# 339501
TYE 563-(CAG)_6_	Exiqon	N/A

**Software and algorithms**		

Incucyte software (version 2019B)	Sartorius	https://www.sartorius.com/en/products/live-cell-imaging-analysis/live-cell-analysis-software/incucyte-software-v2019b
Image Lab™ (Version 6.0.0)	Bio-Rad	https://www.bio-rad.com/en-dk/product/image-lab-software?ID=KRE6P5E8Z
ImageJ (v2.3.0/1.53f51)	(Schindelin et al., 2012)	https://imagej.net/software/fiji/
GraphPad Prism (version 8.4.3 for Windows)	GraphPad	https://www.graphpad.com
IGV genome browser (v2.12.3)	(Thorvaldsdóttir et al., 2013)	https://igv.org/
FastQC (version 0.11.7)	N/A	https://www.bioinformatics.babraham.ac.uk/projects/fastqc/
Trimmomatic (version 0.39)	(Bolger et al., 2014)	https://github.com/usadellab/Trimmomatic
STAR (version 2.7.9a)	(Dobin et al., 2013)	https://github.com/alexdobin/STAR
R (version 4.1.0, 4.1.2, 4.4.1)	R Core Team (2014)	https://www.r-project.org/
DESeq2 (version 1.32.0, version 1.44.0)	R package (Love et al., 2014)	https://github.com/mikelove/DESeq2
apeglm	R package (Zhu et al., 2019)	https://github.com/azhu513/apeglm
Rsubread:featureCounts (version 2.8.1)	R package (Liao et al., 2019)	https://www.bioconductor.org
GSEA (version 4.1.0)	R package (Subramanian et al., 2005)	https://www.gsea-msigdb.org/

**Other**		

3.5 cm dishes	Sarstedt	Cat# 83.3900
Ultra-low attachment dishes	Corning	Cat# 4615
96 well plates	Thermo Fisher	Cat# 167008
13 mm cover glasses	VWR	Cat# 631-1578
24 well plates	Thermo Fisher	Cat# 150628
μ-Slide 8 Well	Ibidi	Cat# 80826
9 mm cover glasses	VWR	Cat# 631-0169
48 well plates	Thermo Fisher	Cat# 150687
18 mm cover glasses	VWR	Cat# 631-0153
12 well plates	Thermo Fisher	Cat# 142475
IncuCyte S3	Essen BioScience	N/A
ChemiDoc MP Imaging System	Bio-Rad	N/A
Laser scanning confocal microscope, LSM800	Zeiss	N/A
Axio Observer.Z1 microscope	Zeiss	N/A
Meso Scale Discovery (MSD) platform	N/A	N/A
NanoDrop One	Thermo Scientific	N/A

### Experimental model and study participant details

#### Sample collection

Skin biopsies were obtained at the Neurology outpatient clinic at the Neuro Center, Neurology, Kuopio University Hospital, Finland from FTD patients diagnosed with bvFTD (Rascovsky et al., 2011) and healthy control individuals. All donors gave written informed consent. In addition, one iPSC line of an FTD patient carrying the C9-HRE was purchased from EBiSC (cell line name: UCLi001-A, Biosamples ID: SAMEA3174431). Detailed information on iPSCs from all donors is provided in Table S1.

#### Ethics statement

The patient data used in this project were pseudonymized and handled using code numbers only as indicated in Table S1. Donors of skin biopsies gave their written informed consent prior to sample collection. The study was performed in accordance with the principles of the declaration of Helsinki. Obtaining skin biopsies, establishment of the biopsy-derived fibroblast cultures, and generation of iPSCs were performed with the permission 123/2016 from the Research Ethics Committee of the Northern Savo Hospital District, Kuopio, Finland.

### Method details

#### Fibroblasts and C9-HRE genotyping

Fibroblast cultures were established from skin biopsies as previously described (Leskelä et al., 2021). Repeat-primed PCR was performed on genomic DNA extracted from corresponding blood samples and skin biopsy-derived fibroblasts to confirm C9-HRE carriership (>60 repeats) and non-carriers (<30 repeats) (Renton et al., 2011). Heterozygous C9-HRE carriership (>145 repeats) was further confirmed in the iPSCs using AmplideX PCR/CE C9orf72 Kit (49581; Asuragen). RNA sequencing data of the iPSC lines confirmed that the sporadic bvFTD did not carry any other FTD-associated mutations. The absence of previously reported sporadic FTD-causing mutations (Mol et al., 2021) from the transcribed RNAs in the iPSC and iMG lines was validated by reviewing their aligned RNA-seq reads at the mutated sites using IGV genome browser (v2.12.3) (Thorvaldsdóttir et al., 2013).

#### iPSC generation and culturing

iPSCs were generated from fibroblasts as described previously (Lehtonen et al., 2018) using CytoTune-iPS Sendai reprogramming kit (A16517, Thermo Fisher). iPSCs (passage 10-40) were maintained on Matrigel coated (356230; Corning) 3.5 cm dishes (83.3900; Sarstedt) in E8 medium (A1517001; Gibco), supplemented with 0.5% [v/v] penicillin/streptomycin [P/S, 15140122; Gibco], 1×E8 supplement [A1517001; Gibco]) at +37°C and 5% CO_2_. Cell culture medium was replaced once per day. Once confluency of 60-80% was reached, iPSCs were split (1:10) by incubating for 3-5 min at +37°C in 0.5 mM EDTA (15575020, Invitrogen) in DPBS (17-512F; Lonza) and kept in 5 μM Y-27632 2HCl (S1049, Selleck Chemicals) containing E8 medium. iPSCs were cryopreserved in E8 medium + 10% (v/v) heat inactivated FBS (hiFBS, 10500; Gibco) + 10% (v/v) DMSO (D2650; Sigma) and stored long-term in liquid nitrogen. iPSCs were confirmed to be free of bacteria, fungi, or mycoplasma. To visualize nuclei, iPSCs were stained with 5 μM Vybrant DyeCycle Green Stain (V35004, Thermo Fisher) for 20 min at +37°C. Images using phase contrast and fluorescence at green channel (300 ms acquisition time) were taken using 10x objective (IncuCyte S3; Essen BioScience). The iPSCs were confirmed by qPCR (*p* = 10-12), IF (*p* = 11-13) and RNA sequencing to express pluripotency and stem cell markers (Figures S1–S3). Detailed information on iPSCs is provided in Table S1 and Figure S17.

#### Differentiation of iPSCs into iMG

One iPSC clone per donor was differentiated into iMG as previously described (Konttinen et al., 2019). In brief, iPSCs were seeded as single cells and kept on Matrigel-coated dishes in E8 Medium supplemented with 25 ng/ml Activin A (120-14E, Peprotech), 5 ng/ml BMP4 (120-05ET, Peprotech), 1 μM CHIR99021 (1386 B8, Axon), and 0.5% (v/v) P/S, and in 5% O2 to induce mesodermal differentiation. The medium was supplemented with 10 μM (day 1; D1) or 1 μM (D2) Y-27632 2HCl, during the first 48 hours. Forty-eight hours after the initiation of differentiation, cells were kept in differentiation medium (DMEM F-12 [21331-020, Gibco] containing 1x GlutaMAX [35050-038, Life Technologies], 0.5% [v/v] P/S [15140-122, Gibco], 64 mg/l ascorbic acid [A8960, Sigma-Aldrich], 14 μg/l sodium selenite [S5261, Sigma-Aldrich], 543 mg/l sodium bicarbonate [25080-094, Thermo Fisher]) and 100 ng/ml FGF2 (AF-100-18B, Peprotech), 10 μM SB431542 (S1067, Selleckchem), 5 μg/ml insulin (I9278, Sigma-Aldrich), and 50 ng/ml VEGF (100-20A, Peprotech) and in 5% O_2_ to induce hemogenic differentiation. On D4-7, cells were cultivated in differentiation medium supplemented with 50 ng/ml FGF2 (AF-100-18B, Peprotech), 5 μg/ml insulin (I9278, Sigma-Aldrich), 50 ng/ml VEGF (100-20A, Peprotech), 50 ng/ml TPO (300-18, Peprotech), 10 ng/ml SCF (300-07, Peprotech), 50 ng/ml IL-6 (200-06, Peprotech), 10 ng/ml IL-3 (200-03, Peprotech), and under 18% O_2_. On D8, erythromyeloid progenitors were transferred to ultra-low attachment dishes (4615, Corning). Medium (IMDM [21980032; Gibco] + 10% [v/v] hiFBS, 0.5% [v/v] P/S, 5 μg/ml insulin [I9278, Sigma-Aldrich], 5 ng/ml MCSF [300-25, Peprotech], 100 ng/ml IL-34 [200-34, Peprotech]) was changed every other day and cell numbers were kept under 3.5 million cells per dish. On D16, iMG were seeded in microglia medium (IMDM [21980032; Gibco] supplemented with 10% [v/v] heat-inactivated FBS [10500; Gibco], 0.5% [v/v] penicillin/streptomycin [15140122; Gibco], 10 ng/ml MCSF [300-25, Peprotech], 10 ng/ml IL-34 [200-34, Peprotech]) on PDL (P6407; Sigma) coated vessels for phagocytosis experiments (96 well plates [167008; Thermo Fisher]), fluorescence *in situ* hybridization (FISH; 13 mm cover glasses [631-1578; VWR] in 24 well plates [150628; Thermo Fisher]), IF (μ-Slide 8 Well [80826; Ibidi], 13 mm cover glasses in 24 well plates, or 9 mm cover glasses [631-0169; VWR] in 48 well plates [150687; Thermo Fisher]), or RNA and protein extraction (18 mm cover glasses [631-0153; VWR] in 12 well plates [142475; Thermo Fisher]). For protein extraction and FISH, erythromyeloid progenitors were frozen on D8, 10, or 12 and stored in liquid nitrogen in freezing medium (10% [v/v] DMSO in microglia medium). After thawing, the differentiation protocol was continued starting from the same day of the protocol on which the cells were frozen (Figure S18).

#### Protein extraction and Western blotting

iMG were seeded on day 16 (100,000 cells/well) and harvested on D22. For experiments with Bafilomycin A1 treatment, iMG were treated on D22 with 150 nM Bafilomycin A1 (B1793-10UG, Sigma Aldrich) for 6 h at +37°C before harvesting. Proteins were extracted, measured in concentration, loaded (15 μg/lane) for SDS-PAGE and transferred as described before (Rostalski et al., 2020). Blots were incubated overnight at +4°C with the following primary antibodies diluted in 1×TBST: anti-C9orf72 (1:500, GTX634482; GeneTex), anti-LAMP2-A (1:1,000, ab18528; Abcam), anti-LC3B (1:3,000, ab51520; Abcam), anti-phospho-TDP-43 (1:3,000, TIP-PTD-P02; CosmoBio), anti-SQSTM1 (1:1,000, 5114S; CST), anti-TDP-43 (1:2,000, 10782-2-AP; Proteintech). Blots were incubated with species-specific horseradish peroxidase-linked secondary antibodies (1:5,000, NA935, NA934V or NA931V; GE Healthcare) for 1 h at RT, and for 5 min with suitable enhanced chemiluminescence substrate solutions (RPN2236 or RPN2235; GE Healthcare; UltraScence Pico Ultra [CCH345; BioHelix]). ChemiDoc MP Imaging System (Bio-Rad) was used to detect the chemiluminescence signals. For each sample, intensity values per area of bands of interest were quantified using Image Lab™ (6.0.0; Bio-Rad). Values were normalized to total protein signals above approx. 18 kDa in size from blots stained with No-Stain Protein Labeling Reagent (A44449, Invitrogen). Data are shown as % of Con_2 line (mean value of each experiment set to 100%).

#### Fixation

Cells were fixed on D22 in 4% (v/v) formaldehyde (28908; Thermo Scientific) in DPBS for 20 min at +37°C, washed twice with DPBS, and stored at +4°C.

#### FISH

FISH was performed similarly as described previously (Pal et al., 2021) using a hybridization temperature of +55°C and fluorescently labeled locked nucleic acid TYE 563-(CCCCGG)_3_ probe to detect the sense foci and TYE 665-(CCCCGG)_2.5_ (339501; Qiagen). TYE 563-(CAG)_6_ probe was used as a negative control probe (Exiqon). H_2_O added to samples was pre-treated with diethyl pyrocarbonate (D5758; Sigma). For quantitative analysis of the sense foci, cells positive for the foci were counted manually from the fluorescence images. FISH for anti-sense foci, microscopy, and image analysis were performed as described (Czuppa et al., 2022).

#### IF and confocal microscopy

For IF, fixed cells were permeabilized using 0.1% (v/v) Triton X-100 in DPBS for 10 min at RT on a rocker and washed 3x with DPBS. Cells were incubated in blocking solution (1.5% or 5% [for C9orf72] [v/v] goat immunoglobulin G isotype control [02-6202; Invitrogen] or 1% [for p62/SQSTM1] or 5% [for TREM2] [w/v] BSA in DPBS) for 30 min at RT on a rocker. Anti-C9orf72 was diluted in 1% (w/v) BSA in DPBS. Other primary and secondary antibodies were diluted in blocking solution and incubated with the cells at RT for 1.5 h or overnight at +4°C followed by washing 3x with DPBS. The following primary antibodies were used: anti-C9orf72 (1:500, GTX634482; GeneTex), anti-CX3CR1 (1:500, 702321; Thermo Fisher), anti-IBA1 (1:250, MA5-27726; Thermo Fisher), anti-LAMP2-A (1:200, ab18528; Abcam), anti-NANOG (1:100, AF1997; R&D Systems), anti-P2RY12 (1:250, HPA014518; Sigma-Aldrich), anti*-*POU5F1 (1:400, MAB4401; EMD Millipore), anti-SSEA4 (1:400, MAB4304; EMD Millipore), anti-SQSTM1 (1:200, sc-28359; Santa Cruz Biotechnology), anti-TDP-43 (1:500, 10782-2-AP; Proteintech), anti-TMEM119 (1:250, HPA051870; Thermo Fisher), anti-TRA-1-81 (1:200, MAB4381; EMD Millipore), anti-TREM2 (1:50, PA5-46980; Thermo Fisher). The following fluorescently labeled species-specific secondary antibodies were used at 1:300 or 1:500 dilution: Alexa Fluor 488 (A-11001, A-11006, A-11034, A-11055, and A-11059; Invitrogen), Alexa Fluor 568 (A-11004, A-11057; Invitrogen), Alexa Fluor 647 (A-21244; Invitrogen). To stain the cell nuclei, cells were mounted in 4′,6-diamidino-2-phenylindole (DAPI)-containing mounting medium (H-1800; Vector Laboratories) or incubated in 0.2 μg/ml DAPI solution (D952, Sigma) in DPBS for 5 min at RT on rocker. For C9orf72, LAMP2-A, p62/SQSTM1 and TDP-43 staining, cells were mounted in phalloidin-containing mounting medium (H-1600; Vector Laboratories). 8-bit images with a resolution of 15.2841 pixels per micron were acquired using laser scanning confocal microscopy (Plan-Apochromat 63x/1.40 Oil DIC M27 objective, Axio Observer.Z1/7, LSM800; Zeiss), or 16-bit images with a resolution of 6.2016 pixels per micron using EC Plan-Neofluar 40x/1.30 Oil DIC M27 objective and Axio Observer.Z1 microscope (Zeiss). Same microscopy settings were used for all samples (including negative control samples where no primary antibody was used) and replication experiments.

#### Image analysis

Confocal microscopy images were processed and analyzed using Fiji (2.3.0/1.53f51; (Schindelin et al., 2012)). For cell body and nucleus segmentation, phalloidin images were used to create masks, outlining the cell bodies and allowing measurement of the total cell body area per image. For this, auto local thresholding (version 1.10, Sauvola method, radius = 15, default *k* and *r* values) was applied, followed by Gaussian Blur filter (sigma = 2 [sigma = 4 for C9orf72 images]) and removal of particles with a radius smaller than 5 μm. To fill holes within cell bodies, particles of a size between 0-4 μm were included. After watershed segmentation, regions of interest were outlined without further area or shape exclusion. DAPI images were used to indicate nuclei and measure nuclear areas per image (for TDP-43 analysis). To segment nuclei, background subtraction (rolling ball radius = 100 pixels), Gaussian Blur filter (sigma = 2), auto thresholding (Renyi’s entropy method), and removal of particles with a radius smaller than 5 μm were applied followed by fill holes command.

C9orf72 and TDP-43 signals were quantified as sum intensities of the fluorescence signal originating from the fluorescently labeled secondary antibody within phalloidin-positive areas per image. Sum intensities were normalized to phalloidin-positive area per image. Values from samples without primary antibody incubation (negative control) were subtracted from values from samples with primary antibody incubation.

For TDP-43 translocation analysis, images were analyzed as described here (Rostalski et al., 2020). In brief, TDP-43 signals were quantified as sum intensities of the fluorescence signal originating from the secondary antibodies in nuclear (DAPI-positive) and cytosolic areas (nuclear signal subtracted from signal within whole cell body [phalloidin-positive areas]) for each image. Sum intensities were normalized to nuclear and cytosolic area sizes, respectively for each image. To determine unspecific signal intensities for each cell line, intensity values for nucleus and cytosol were obtained from samples stained without primary antibody (negative control). Maximum unspecific signal intensities for each cell line per experiment were calculated. Cytosolic to nuclear TDP-43 signal ratio was calculated by subtracting the maximum unspecific area-normalized sum intensities for each line from nuclear and cytosol signals, respectively. To determine TDP-43 translocation into the cytosol, a recently developed TDP-43 translocation analysis was used. In brief, in this analysis the signal of nuclear and cytosolic TDP-43 is categorized into four stages (none, mild, moderate, severe TDP-43 translocation from nucleus to cytosol; Figure S12A). For this, the number of translocation-positive cells was manually counted based on nuclear and cytosolic TDP-43 intensity values. For the manual analysis, TDP-43 stages “none” and “mild” were grouped as “non-pathological” and “moderate” and “severe” as “pathological” because the cytosolic to nuclear TDP-43 intensity signals did not differ between “none” and “mild” nor “moderate” and “severe” groups (Figure S12B) but differed between the groups “non-pathological” and “pathological” (Figure S12C).

For p62/SQSTM1 and LAMP2-A particle analysis, signals were quantified as sum intensities of the fluorescence signal originating from the secondary antibody normalized to p62/SQSTM1- or LAMP2-A-positive particle areas. Mean size and number of p62/SQSTM1- or LAMP2-A positive particles per total cell body area within phalloidin-positive areas per image were calculated. Background subtraction (rolling ball radius = 10 pixels) and thresholding (Maximum Entropy algorithm [p62/SQSTM1]; Renyi’s entropy method [LAMP2-A]) were applied so that a minimal number of particles were detected in samples stained without primary antibody (negative control). Only particles with a minimum area of 0.5 μm^2^ were included in the analysis (p62/SQSTM1).

For IBA1 intensity analysis, individual nuclei were detected as DAPI-positive particles, with a minimum area of 5 μm^2^. Background subtraction (rolling ball radius = 100 pixels), thresholding (Renyi’s entropy method) and filling of holes were applied prior to nuclear detection. Each nuclear region of interest was stretched to 1.6 times the original size. IBA1 signal was quantified as sum intensities of the fluorescence signal originating from the fluorescently labeled secondary antibody within the stretched nuclear areas per image. Sum intensities were normalized to the stretched nuclear area per cell. Cells with IBA1 sum intensity higher than the mean sum intensity in samples stained without primary antibody (negative control) were considered IBA1-positive.

#### Phagocytosis assay

On D16, iMG were plated on 96-well plates (approx. 30,000 cells/well). For serum starvation, 24 h prior to assay, iMG were kept in serum-free microglia medium. On D23 or 24, cells were incubated in serum-free microglia medium supplemented with zymosan-coupled pH-sensitive fluorescent particles (P35364, Invitrogen) at final concentrations of 62.5 μg/ml. Cells without particles were used for background subtraction in the red channel. Microscopy images were acquired with 20x objective from brightfield, and red fluorescent channels (300 ms acquisition time) every 30 min using IncuCyte S3 (Essen BioScience). Per experiment, four images per well per biological replicate (3-4) per line were acquired. For analysis, the IncuCyte software (version 2019B) was used. For bright field channel, segment adjustment of 0.5 was used. For the red channel, Top-Hat segmentation (radius 10 μm, 2 RCU threshold) and edge sensitivity of -60 were used. To estimate the phagocytic activity, the size of the red fluorescent area within cells or intensity values of red channel (Total Red Integrated Intensity [RCU x μm^2^/image]) were calculated and normalized to total cell area (before beads were added) for each image. Values were calculated for each biological replicate and pooled from two independent differentiations.

#### DPR immunoassay

iMG were seeded on D16 (400,000-500,000 cells/well) and snap frozen on dry ice after removing medium on D23. Sample preparation and the DPR immunoassay were carried out on the Meso Scale Discovery (MSD) platform (Czuppa et al., 2022). Total protein levels in all samples were used to normalize the signals. In total, one control iMG line (Con_3) and two sporadic iMG lines (Ftd_1, Ftd_4) as C9-HRE non-carrying controls, and all C9-HRE iMG lines (Ftd_5, Ftd_6, Ftd_7) were included in the immunoassay.

#### RNA extraction

iPSCs were detached with EDTA, centrifuged for 10 min at 8,000 x g, resuspended, and processed in NucleoProtect RNA (740400.250, MACHEREY-NAGEL) as described by the manufacturer, and stored at -80°C until RNA extraction. On D16, iMG were plated (225,000 cells/well). On D23, iMG were washed twice with ice-cold DPBS and scraped in 200 μl ice-cold DPBS. RNA extraction was conducted directly. Total RNA was isolated (11828665001, Roche Molecular Systems, Inc.), and RNA concentrations were measured using NanoDrop One (Thermo Scientific).

#### RNA sequencing

Bulk RNA sequencing (RNA-seq) was performed using RNA extracted as described above from iPSCs and iMG. Library preparation and RNA sequencing was conducted by Novogene (UK) Company Limited as described previously (Wittrahm et al., 2021) and yielded 19.9–32.4 million sequenced fragments per sample.

### Quantification and statistical analyses

#### RNA sequencing analysis

The 150 nt pair-end RNA-seq reads were quality controlled using FastQC (version 0.11.7) (https://www.bioinformatics.babraham.ac.uk/projects/fastqc/). Reads were then trimmed with Trimmomatic (version 0.39) (Bolger et al., 2014) to remove Illumina sequencing adapters and poor quality read ends, using as essential settings: ILLUMINACLIP:2:30:10:2:true, SLIDINGWINDOW:4:10, LEADING:3, TRAILING:3, MINLEN:50. Reads aligning to mitochondrial DNA or ribosomal RNA, phiX174 genome, or composed of a single nucleotide, were removed using STAR (version 2.7.9a) (Dobin et al., 2013). The remaining reads were aligned to the Gencode human transcriptome version 38 (for genome version hg38) using STAR (version 2.7.9a) (Dobin et al., 2013) with essential non-default settings: --seedSearchStartLmax 12, --alignSJoverhangMin 15, --outFilterMultimapNmax 100, --outFilterMismatchNmax 33, --outFilterMatchNminOverLread 0, --outFilterScoreMinOverLread 0.3, and --outFilterType BySJout. The unstranded, uniquely mapping, gene-wise counts for primary alignments produced by STAR were collected in R (version 4.1.0) using Rsubread::featureCounts (version 2.8.1) (Liao et al., 2019), ranging from 14.7 to 24.3 million per sample. After normalization, using varianceStabilizingTransformation (from DESeq2 version 1.32.0), the data were subjected to sample-level quality control and a minor batch effect was identified between sequencing batches (Figure S19). Batch-corrected DEGs between experimental groups were identified in R (version 4.1.2) using DESeq2 (version 1.32.0) (Love et al., 2014) by employing Wald statistic and lfcShrink for FC shrinkage (type=“apeglm”) (Zhu et al., 2019). Pathway enrichment analysis was performed on the gene lists ranked by the pairwise DEG test log2FCs using command line GSEA (GSEA version 4.1.0) (Subramanian et al., 2005) with Molecular Signatures Database gene sets (MSigDB, version 7.5.1) (Liberzon et al., 2011, 2015; Subramanian et al., 2005). To validate the microglial identity of the iMG cells, raw RNA-seq read count matrices of Vahsen et al. (2022) (GSE200037), Abud et al. (2017) (GSE89189) and Muffat et al. (2016) (GSE85839) studies were downloaded, integrated with the raw iMG read counts via gene IDs, and converted to DESeqDataSet format with ‘design = groupid’ where groupid refers to cell type as indicated in the source data. The integrated data were normalized in R (version 4.4.1) using varianceStabilizingTransformation (from DESeq2 version 1.44.0) in the non-blind mode (blind = FALSE), to account for differences in gene-wise variance between cell types.

#### Statistical analyses

Shapiro-Wilk test was used to test for normal distribution. Bartlett’s test was used to test for equality of variances. To test for significance between two experimental groups, two-tailed independent samples t-test or Mann-Whitney U test was performed, for more than two groups, one-way analysis of variance (ANOVA) followed by Tukey’s multiple comparisons test or Sidak’s multiple comparisons test (phagocytosis assay) was used if data points were normally distributed and variances equal between groups. Otherwise Kruskal-Wallis test followed by Dunn’s multiple comparisons test was used. Two-way ANOVA followed by Sidak’s multiple comparisons test was used for testing statistical significance related to treatment effect (Bafilomycin A1). To assess statistical independence between categorical variables, Chi-Square test was used. For tests and data visualization, GraphPad Prism (version 8.4.3 for Windows, GraphPad Software, San Diego, California USA, www.graphpad.com) was used. Test statistics are reported in the figure legends. *p*-values for pairwise comparisons are indicated above the compared groups within the figures. *p-*values <0.05 were considered significant. Number of independent differentiations of iMG from iPSCs, statistical tests and results, and descriptive statistics are indicated in each figure legend. Average of values per patient line and differentiation are represented as individual data points (“*n*”). Microscopy images shown in the manuscript were processed using ImageJ (1.53f51).

## Supplemental information

Document S1. Figures S1–S19 and Table S1

Table S2. DEG and GSEA results on RNA sequencing data

Document S2. Article plus supplemental information
