# Supplementary material for: C9orf72-associated and sporadic FTD patient iPSC-microglia show differences in phagocytosis and gene expression
Source: Stem Cell Reports. 2026 Aug 6;21(9):103026. doi: 10.1016/j.stemcr.2026.103026 (PMC13555443; doi:10.1016/j.stemcr.2026.103026)
Supplement: Supplementary file 1 [file mmc1.pdf]

Stem Cell Reports, Volume *21*

Supplemental Information

C9orf72-associated and sporadic FTD patient iPSC-microglia show differences in phagocytosis and gene expression

Hannah Rostalski, Tomi Hietanen, Dorit Hoffmann, Sami Heikkinen, Nadine Huber, Ashutosh Dhingra, Salvador Rodriguez-Nieto, Teemu Kuulasmaa, Sohvi Ohtonen, Henna Jäntti, Viivi Pekkala, Stina Leskelä, Petra Mäkinen, Kasper Katisko, Päivi Hartikainen, Šárka Lehtonen, Eino Solje, Jari Koistinaho, Tarja Malm, Anne M. Portaankorva, Teemu Natunen, Henna Martiskainen, Mari Takalo, Mikko Hiltunen, and Annakaisa Haapasalo

Supplemental Information (SI)

Supplemental Figures

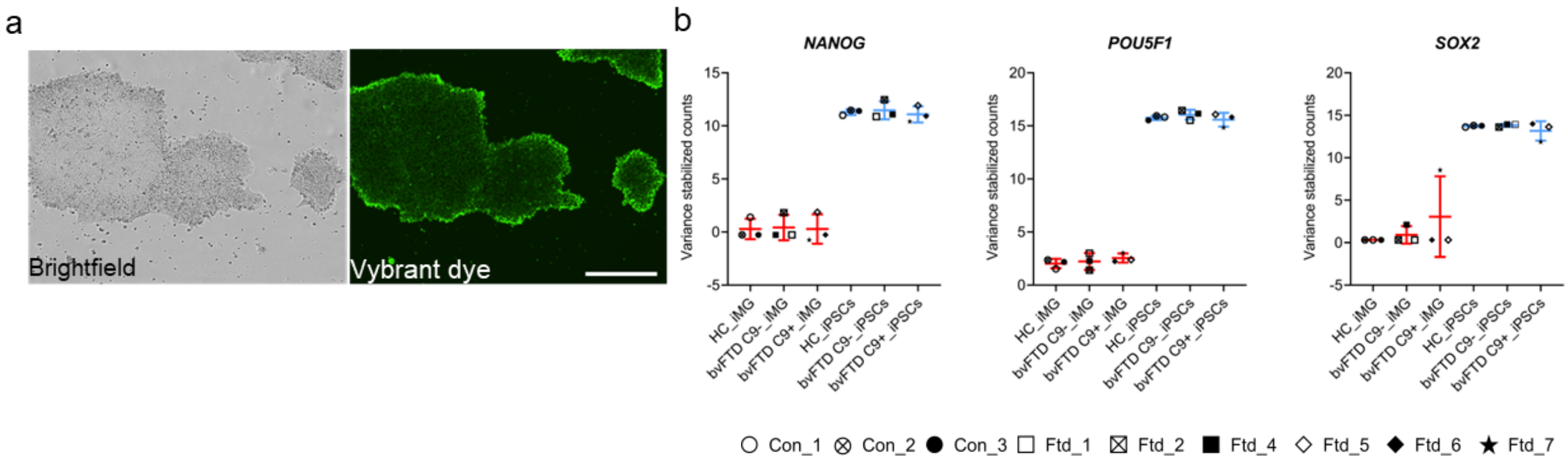

Figure S1. Quality control of iPSCs from healthy controls, sporadic (C9-) and C9-HRE-carrying bvFTD (C9+) patients. (a) A representative image of iPSCs from which the iMG were generated in this study. iMG were incubated with Vybrant dye (nuclear stain; green). Scale bar = 400 µm. (b) RNA sequencing data (mean ± standard deviation of variance stabilized counts) show expression of different stem cell marker genes in iPSCs (blue) vs. in the differentiated iMG (red) for each donor. Data on iMG RNA are shown from one differentiation batch; each data point represents one RNA sample per donor. The individual iMG lines are indicated by different symbols at the bottom.

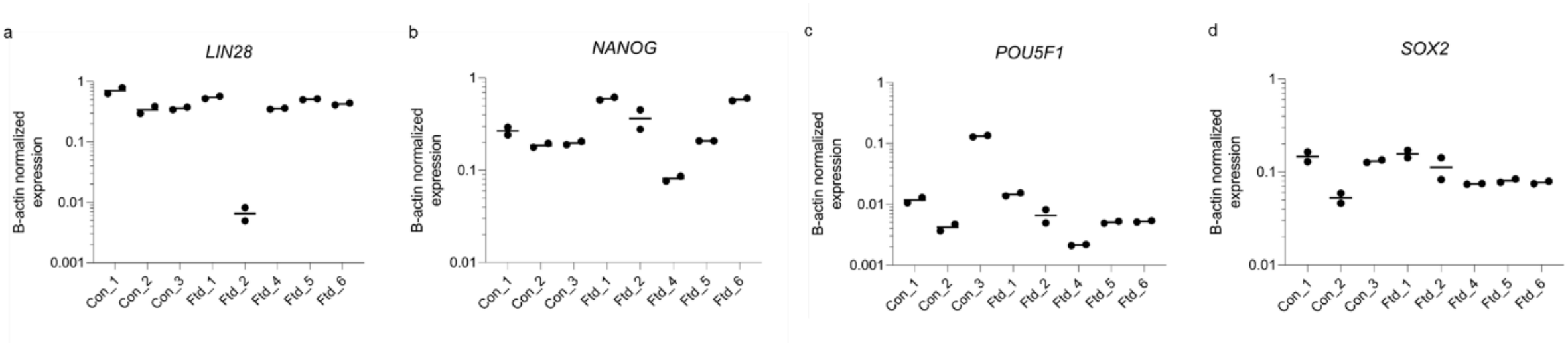

Figure S2. iPSCs from healthy controls, sporadic (C9-) and C9-HRE-carrying (C9+) bvFTD patients express pluripotency genes. qPCR was used to assess gene expression levels of different pluripotency marker genes in iPSCs. Each datapoint indicates technical replicates in each iPSC line.

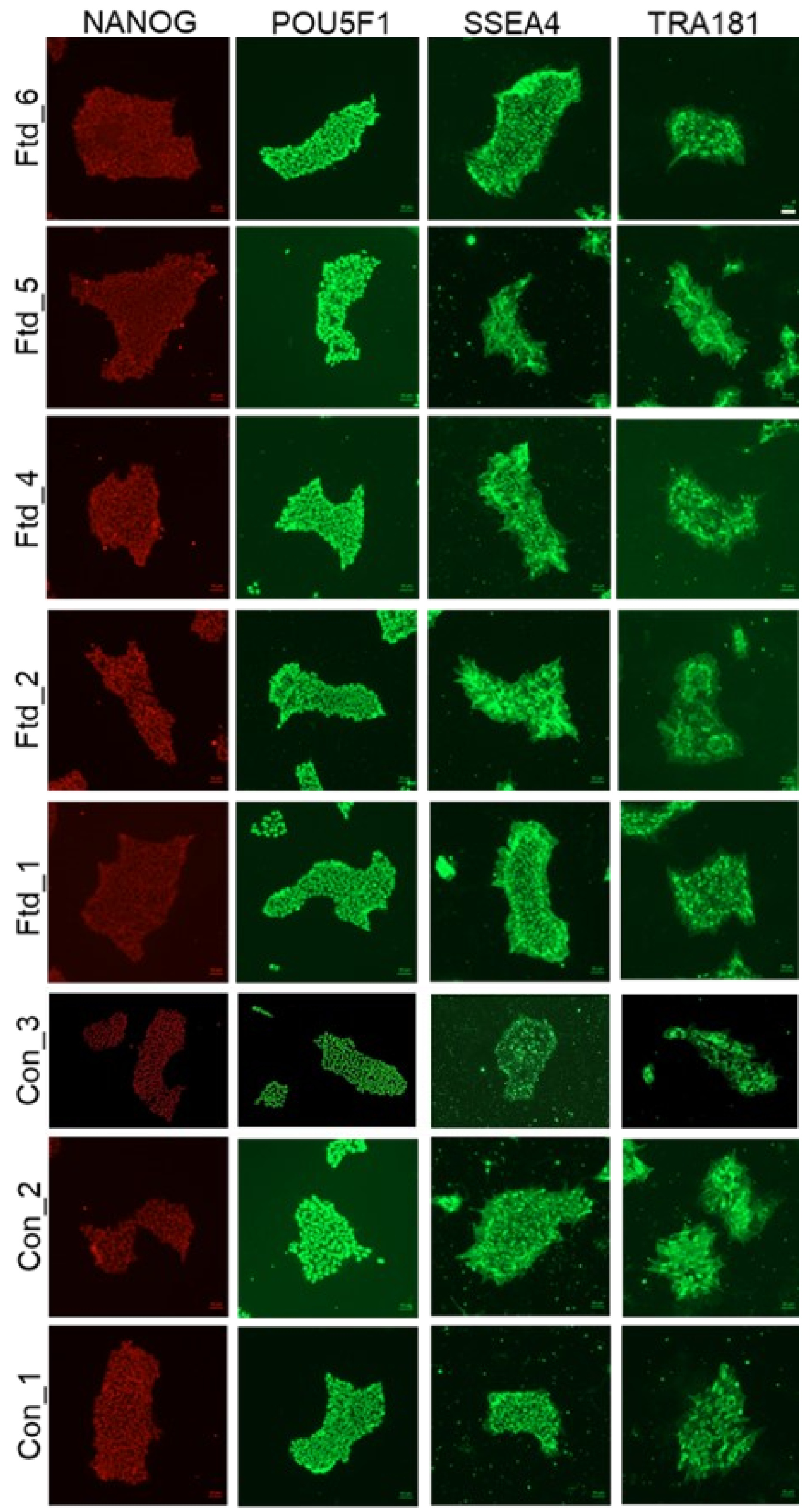

Figure S3. iPSCs from healthy controls, sporadic (C9-) and C9-HRE-carrying bvFTD (C9+) patients express pluripotency markers *NANOG*, *POU5F1, SSEA4,* and *TRA181*. iPSCs were stained for different pluripotency markers. Scale bar = 50 µm. Image adjustments in PowerPoint: all, brightness +20% (except for Con_3 brightness +40%).

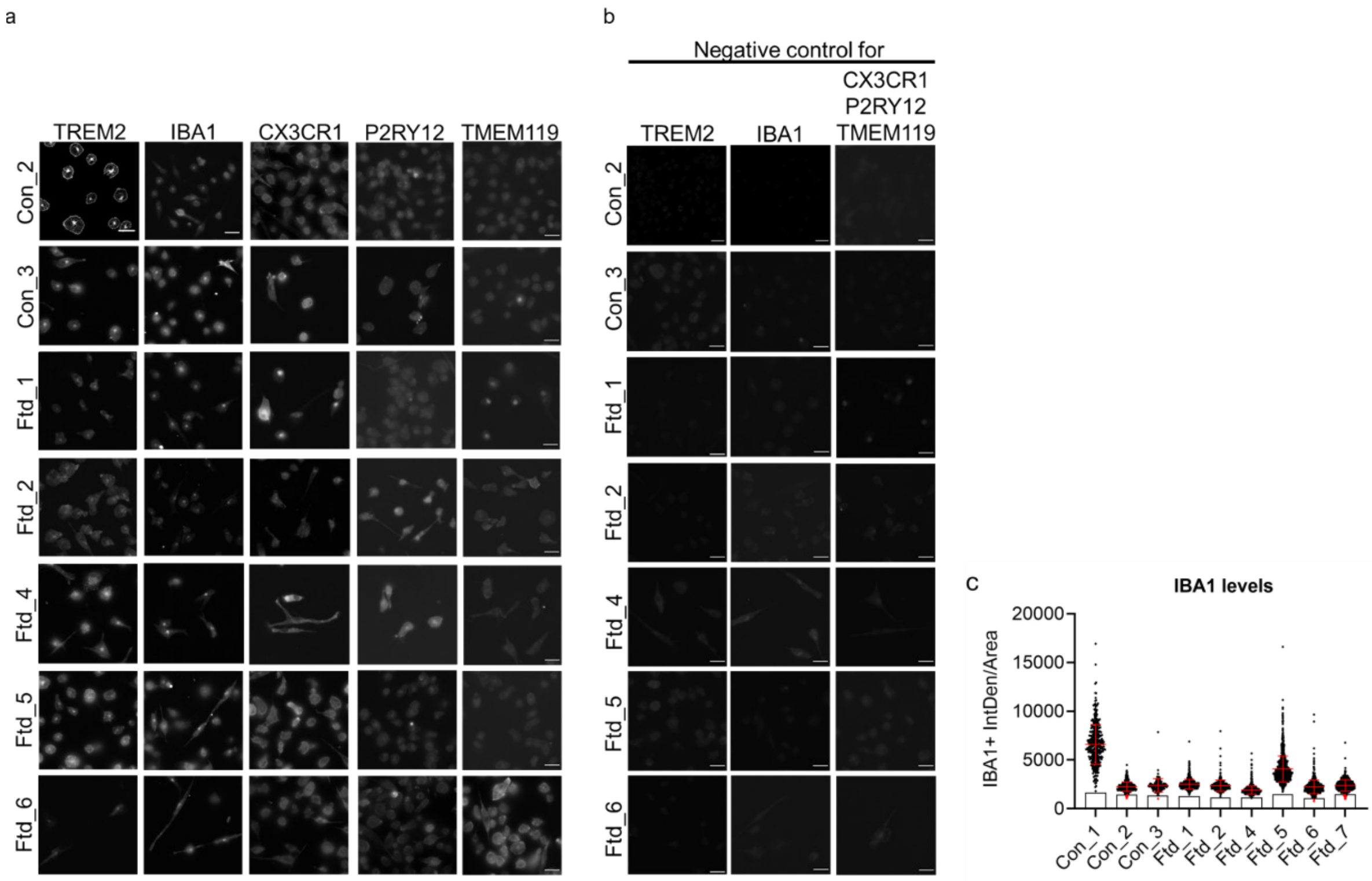

Figure S4. Myeloid/microglial markers in iMG from healthy controls, sporadic (C9-) and C9-HRE-carrying (C9+) bvFTD patients, related to Figure 1. (a) iMG were stained for myeloid lineage/microglial marker proteins. (b) Negative control staining without primary antibodies (secondary antibody only). (c) Quantification of IBA1 levels based on immunofluorescence images. Each data point represents IBA1 levels from one cell; *n* [Con_1] = 379; *n* [Con_2] = 390; *n* [Con_3] = 123; *n* [Ftd_1] = 1004; *n* [Ftd_2] = 371; *n* [Ftd_4] = 1389; *n* [Ftd_5] = 1512; *n* [Ftd_6] = 775; *n* [Ftd_7] = 468. The bars show mean fluorescence intensity of negative controls from *n* cells; *n* [Con_1] = 153; *n* [Con_2] = 221; *n* [Con_3] = 36; *n* [Ftd_1] = 399; *n* [Ftd_2] = 252; *n* [Ftd_4] = 681; *n* [Ftd_5] = 456; *n* [Ftd_6] = 296; *n* [Ftd_7] = 214. Percentage of iMG expressing IBA1 levels higher than the mean of the negative controls was 100%, except in Con_2 (382/390; 97.95%), Con_3 (122/123; 99.19%), Ftd_6 (772/775; 99.61%) and Ftd_7 (455/468; 97.22%). Data points below the mean of the negative controls have been colored in red. Scale bar = 25 µm. No image adjustments were made in any of the images. Data are shown from one differentiation batch.

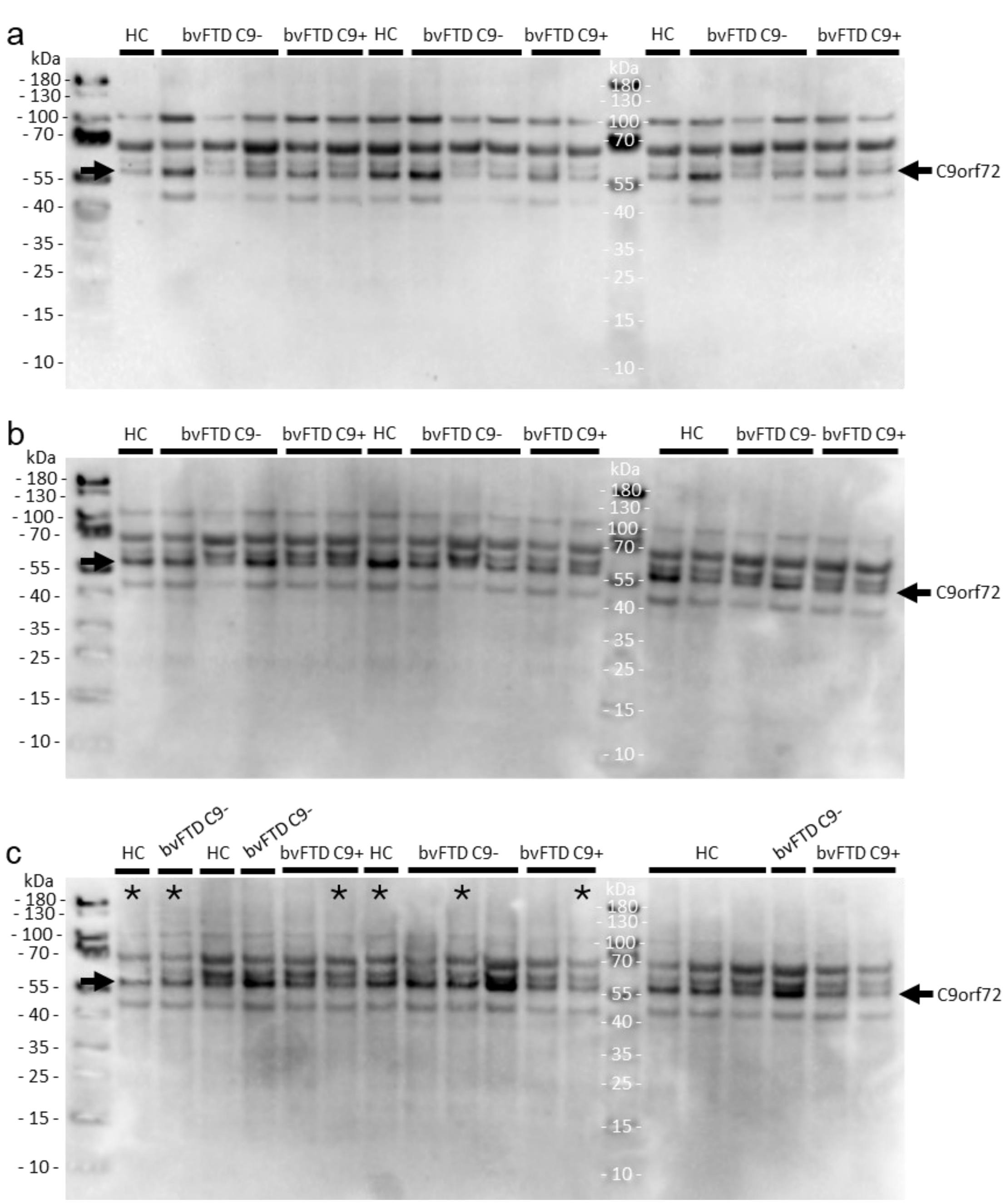

Figure S5. C9orf72 Western Blots from iMG of healthy controls, sporadic (C9-) and C9-HRE-carrying (C9+) bvFTD patients, related to Figure 2. (a-c) Uncropped Western blot images (showing C9orf72 signals) are shown from three independent differentiation batches for individual iMG lines. The quantified C9orf72 bands have been marked with arrows. Asterisks indicate cropped lanes (2, 3, 7, 8, 10, 13 from blot c) shown in Figure 2.

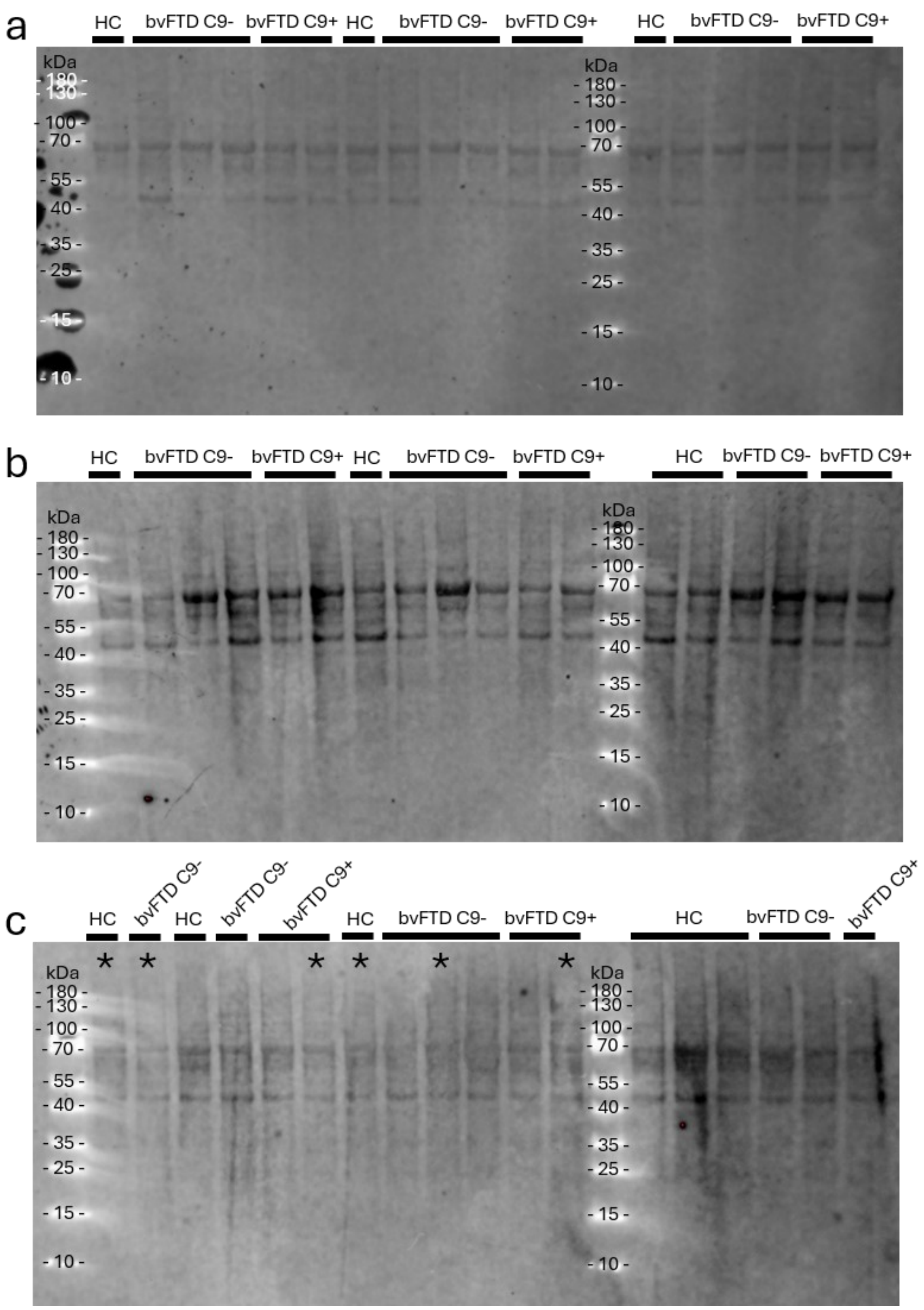

Figure S6. Total protein-stained Western blots from iMG of healthy controls, sporadic (C9-) and C9-HRE-carrying (C9+) bvFTD patients, related to Figure 2. (a-c) Uncropped Western blot images (showing No-Stain™ Protein Labeling Reagent signals) are shown from three independent differentiation batches for individual iMG lines. Asterisks indicate cropped lanes (2, 3, 7, 8, 10, 13 from blot c) shown in Figure 2.

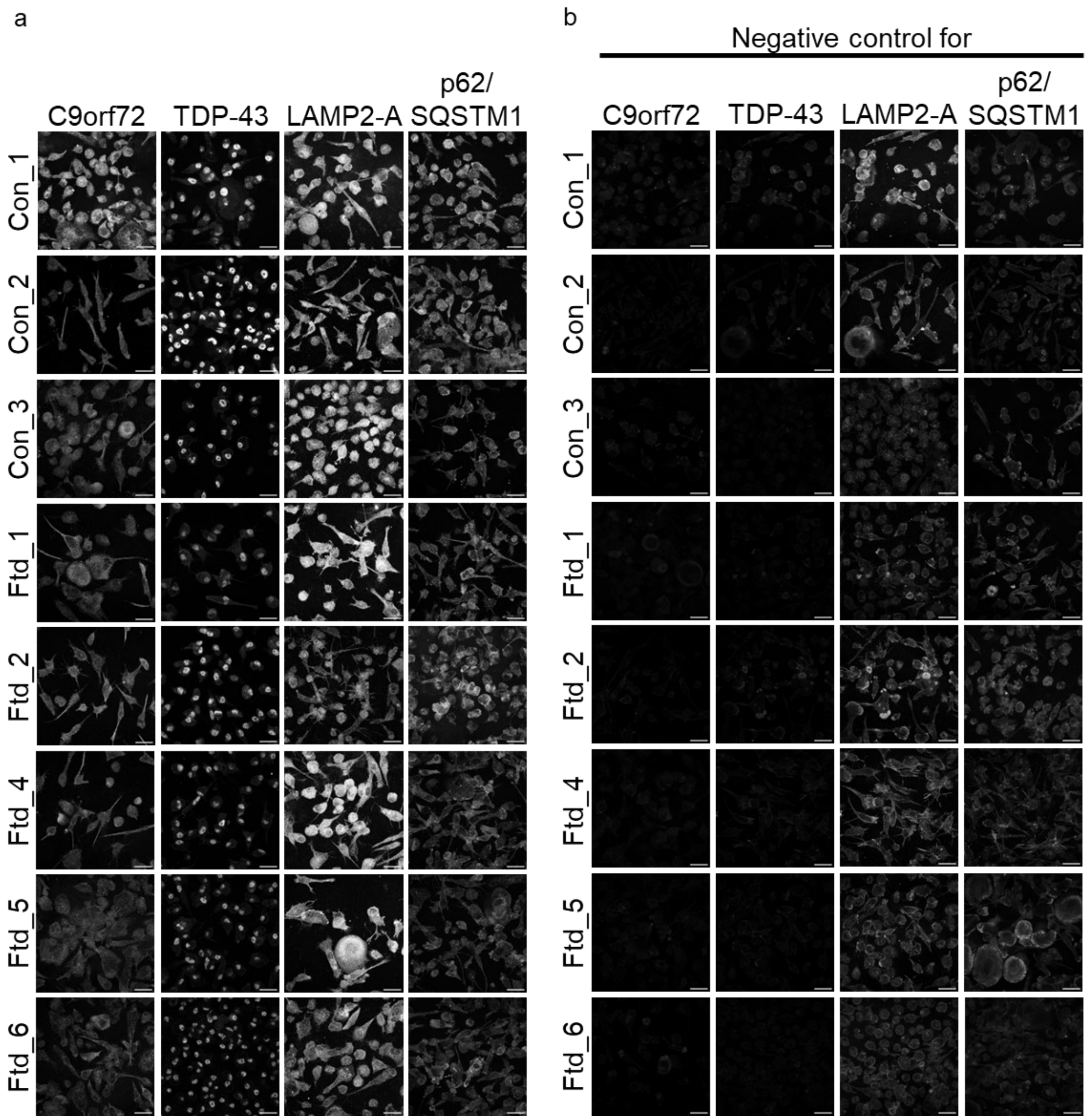

Figure S7. Representative images of C9orf72, TDP-43, LAMP2-A and p62/SQSTM1 staining in iMG from healthy controls, sporadic (C9-) and C9-HRE-carrying (C9+) bvFTD patients; related to Figure 2, 3, 5. (a) Primary antibody staining in iMG for C9orf72, TDP-43, LAMP2-A and p62/SQSTM1. (b) Negative control staining without primary antibodies (secondary antibody only). Scale bar = 30 µm. Image adjustments in PowerPoint: all C9orf72, brightness +40%, contrast -20%; all TDP-43, brightness +30%; all LAMP2-A, brightness +80%; all p62/SQSTM1, brightness +50%. Data are shown from one differentiation batch.

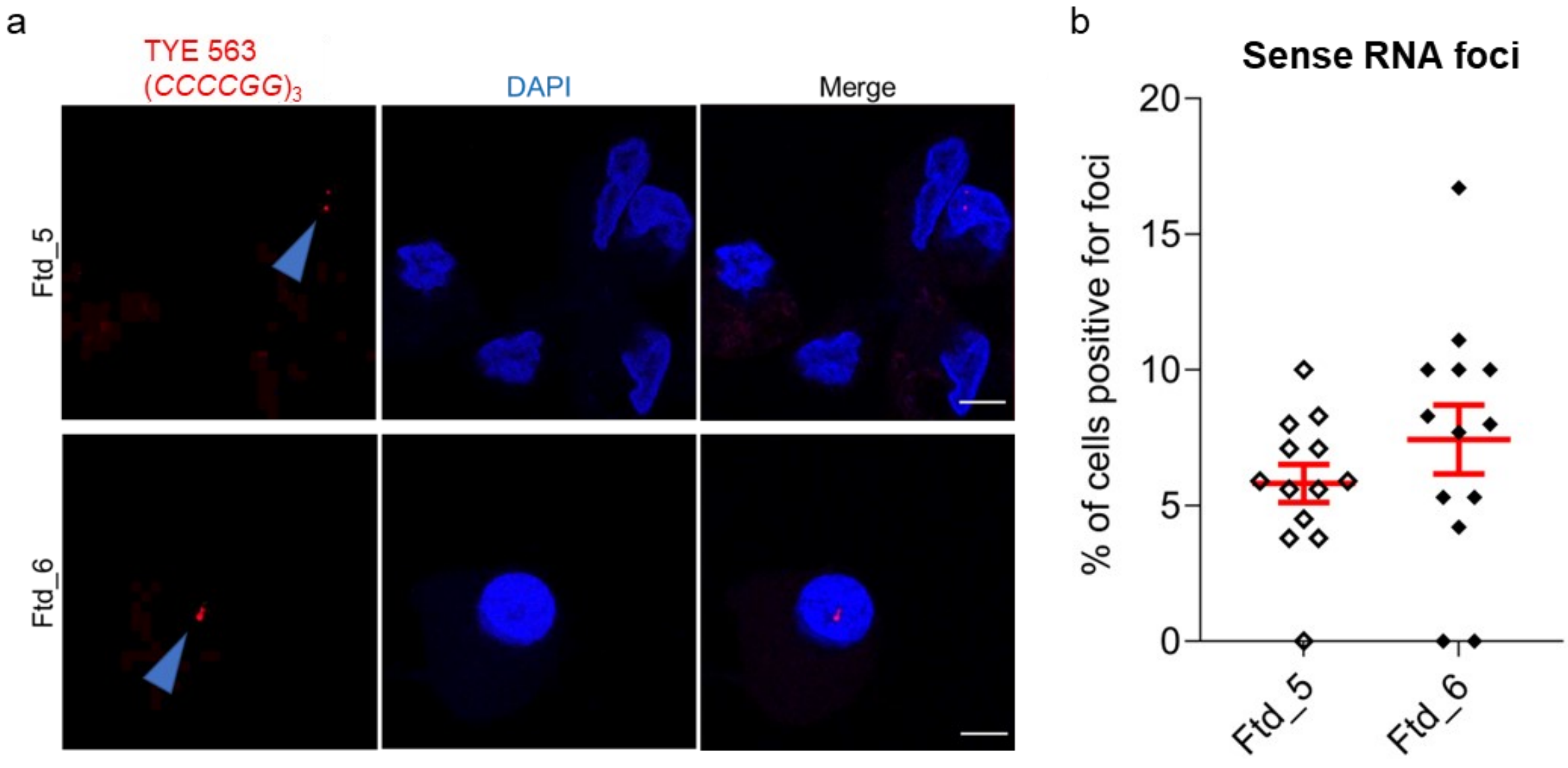

Figure S8. Assessment of sense RNA foci in the iMG of two C9-HRE-carrying (C9+) bvFTD patients, related to Figure 2. (a) Microscopy images showing intranuclear sense RNA foci, detected by fluorescence *in situ* hybridization (FISH) using the TYE 563(CCCCGG)$_3$ probe (red) and indicated by the blue arrow. Nuclei were counterstained with DAPI (blue). Data are shown from one differentiation batch. Scale bar = 5 µm. Image adjustments in PowerPoint: all probe, brightness +80%; all DAPI, brightness +75%; all merge, brightness +30%. (b) Quantification of sense foci for each line. Each data point represents one image. A total of 9-34 cells were counted per image. All data are from one differentiation batch. Descriptive statistics are shown as mean ± standard error mean.

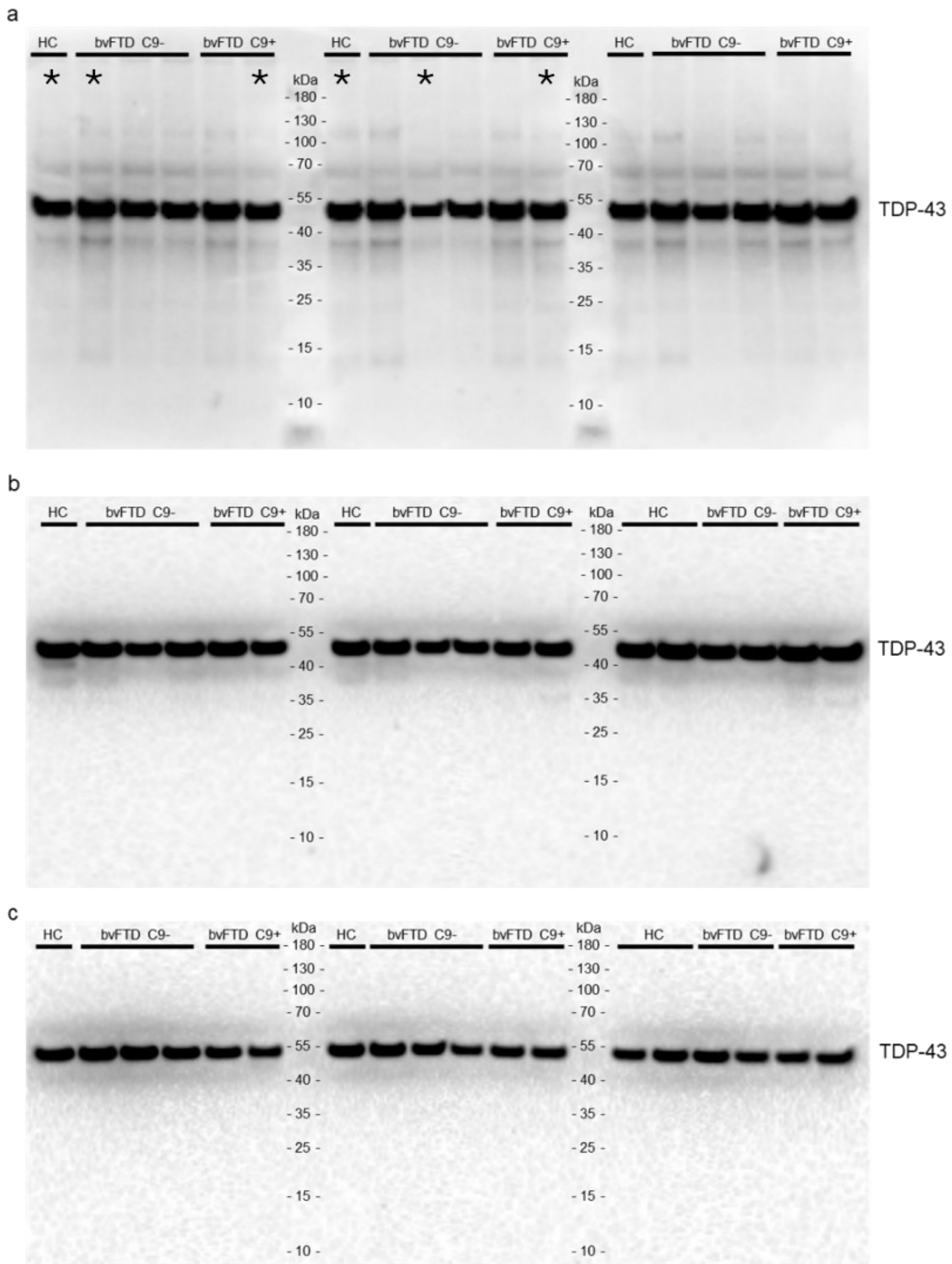

Figure S9. TDP-43 Western Blots from iMG of healthy controls, sporadic (C9-) and C9- HRE-carrying (C9+) bvFTD patients, related to Figure 3. (a-c) Uncropped Western blot images (showing total TDP-43 signals) are shown from three independent differentiation batches for individual iMG lines. Asterisks indicate cropped lanes (1, 2, 6, 8, 10, 13 from blot a) shown in Figure 3. Signal was enhanced after image acquisition to check for faint signals emerging from potential C-terminal fragments. The presence of C-terminal TDP-43 fragments (~35 and ~25 kDa) was not evident on the blots.

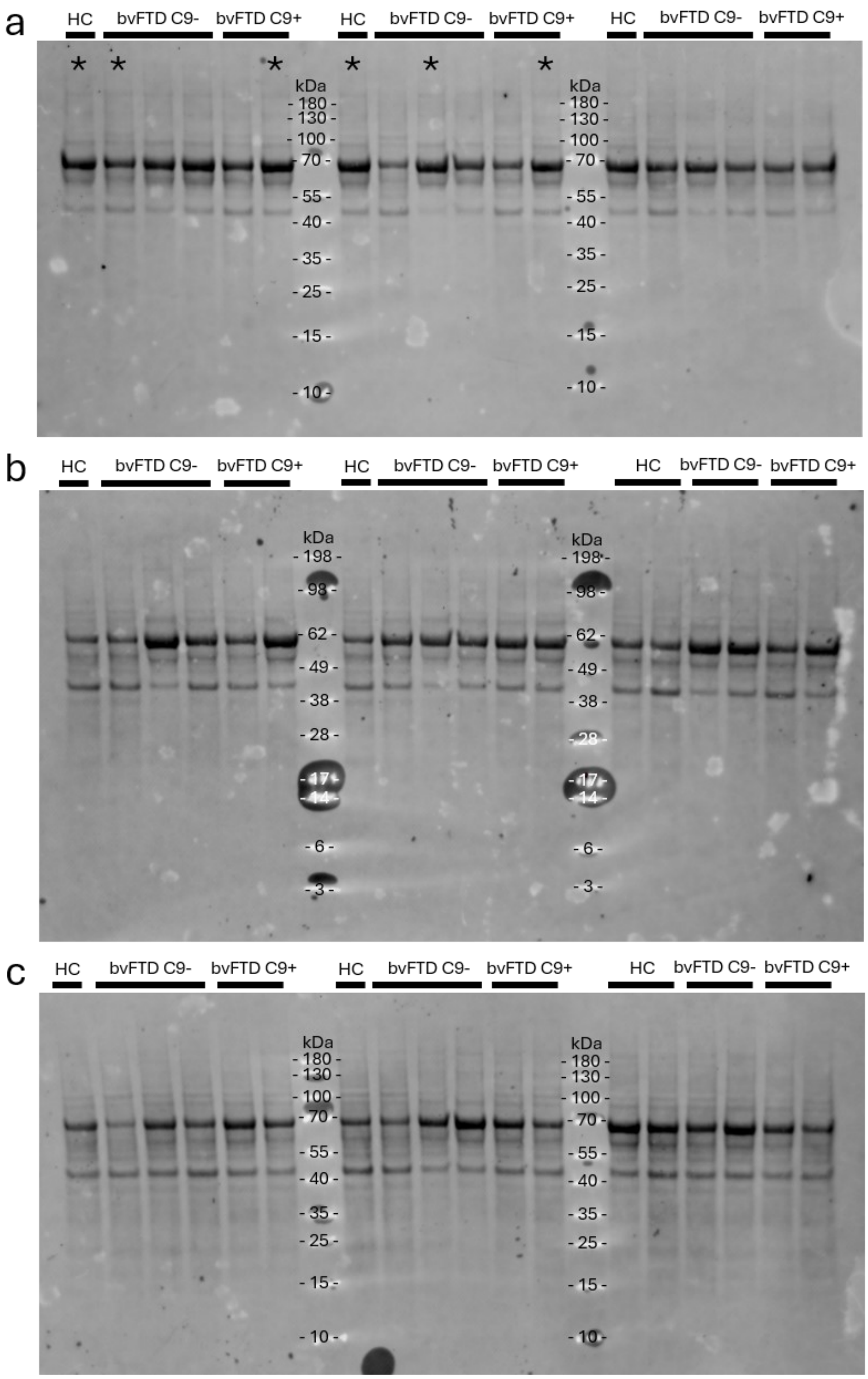

Figure S10. Total protein-stained Western blots from iMG of healthy controls, sporadic (C9-) and C9-HRE-carrying (C9+) bvFTD patients, related to Figures 3-4. (a-c) Uncropped Western blot images (showing No-Stain™ Protein Labeling Reagent signals) are shown from three independent differentiation batches for individual iMG lines. Asterisks indicate cropped lanes (1, 2, 6, 8, 10, 13 from blot a) shown in Figure 3-4.

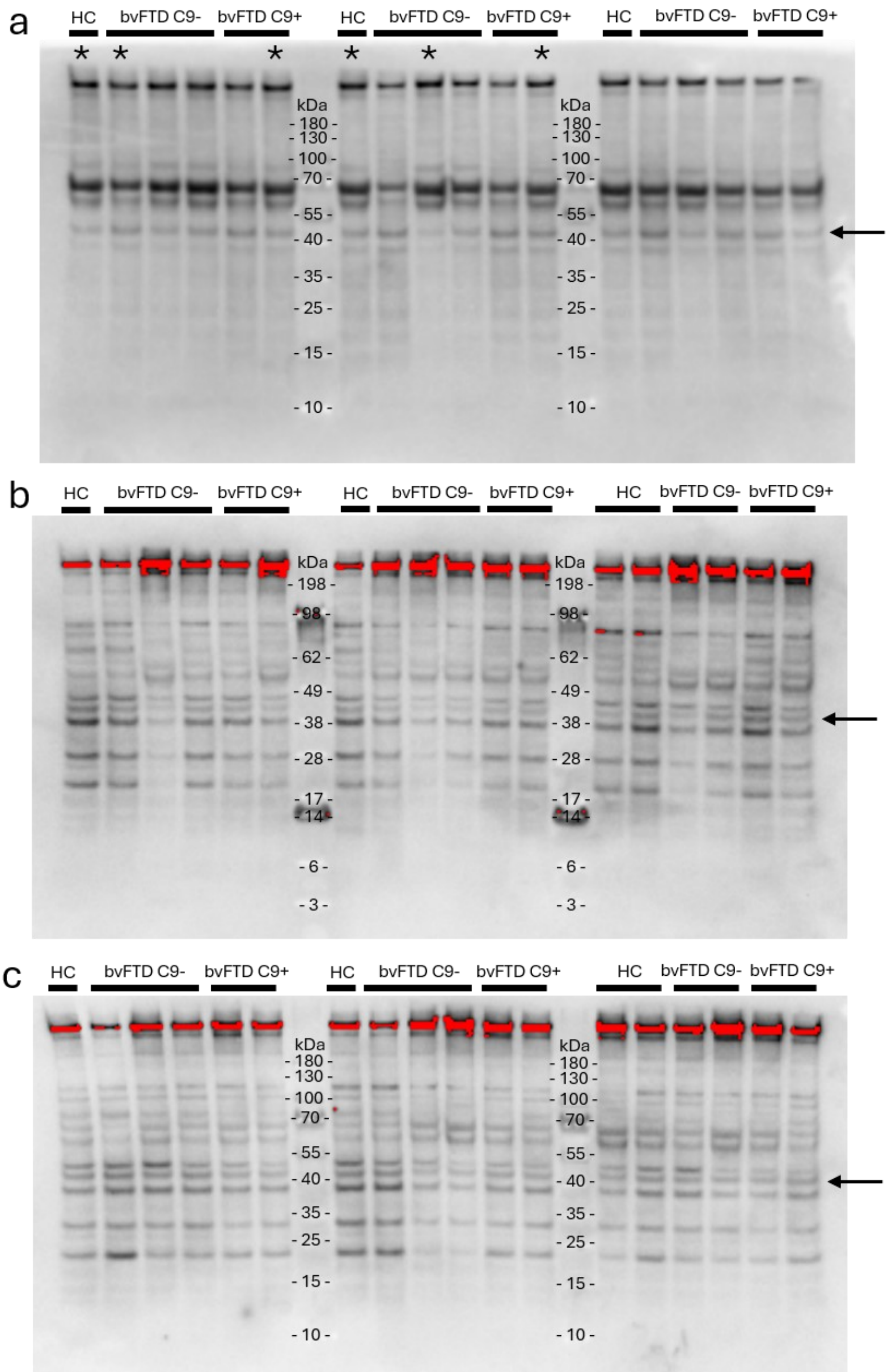

Figure S11. pTDP-43 Western Blots from iMG of healthy controls, sporadic (C9-) and C9- HRE-carrying (C9+) bvFTD patients, related to Figure 3. (a-c) Uncropped Western blot images (showing pTDP-43 signals) are shown from three independent differentiation batches for individual iMG lines. The quantified pTDP-43 bands have been marked with arrows. Asterisks indicate cropped lanes (1, 2, 6, 8, 10, 13 from blot a) shown in Figure 3.

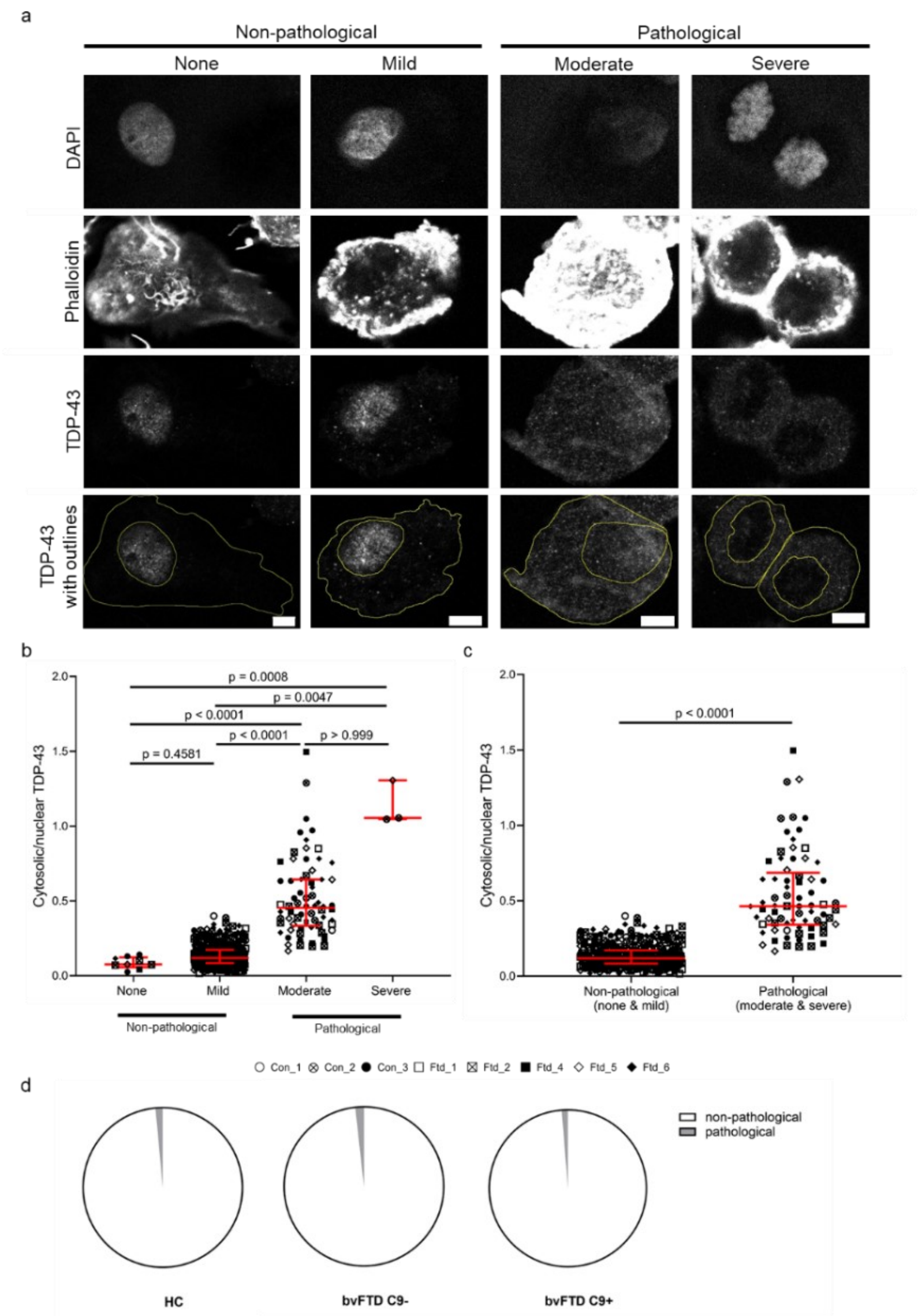

Figure S12. TDP-43 translocation analysis in iMG, related to Figure 3. (a) Representative microscopy images of TDP-43 translocation types are shown. Yellow outlines depict areas of DAPI-positive nuclei and phalloidin-positive areas, indicating cell boundaries. Scale bar = 5 µm. For statistical testing, Kruskal-Wallis test followed by Dunn's multiple comparisons test (b; H = 170.7, p < 0.0001, n [type 1] = 9, n [type 2] = 391, n [type 3] = 70, n [type 4] = 3) and two-tailed Mann-Whitney test (c; test statistics = 712, n [non-pathological] = 400, n [pathological] = 73) were used; *n* = number of individuals cells. (d) Similar ratios of non-pathological *vs.* pathological TDP-43 translocation status were detected in healthy control iMG (98.539% vs. 1.461%), sporadic bvFTD iMG (98.277% vs. 1.723%), and C9-HRE-carrying bvFTD iMG (98.759% vs. 1.241%), respectively, without significantly different distribution (p = 0.3630, χ^2 = 2.027, degree of freedom = 2). Data are shown from three independent differentiation batches. The individual iMG lines are indicated by different symbols at the bottom.

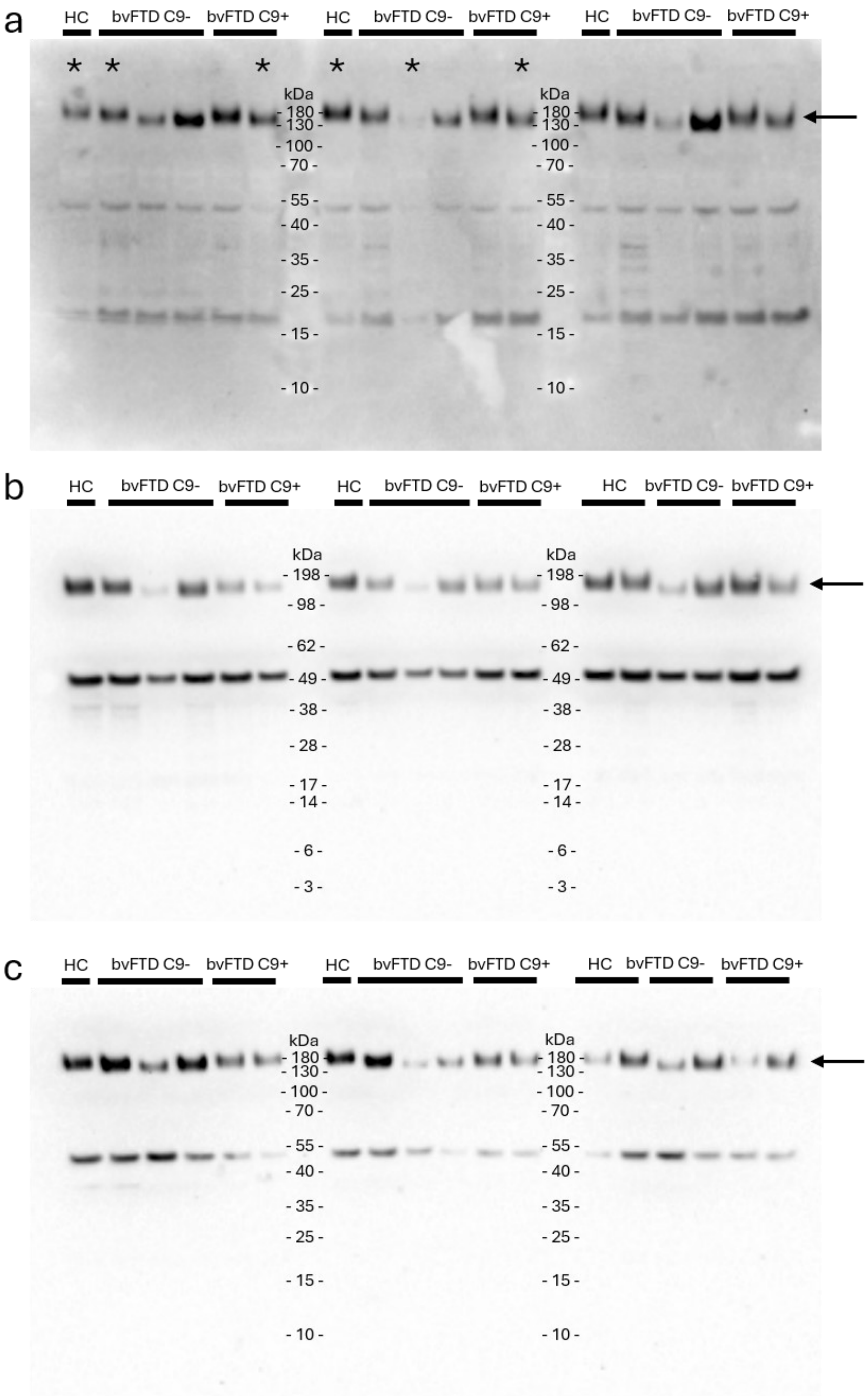

Figure S13. LAMP2-A Western Blots from iMG of healthy controls, sporadic (C9-) and C9- HRE-carrying (C9+) bvFTD patients, related to Figure 4. (a-c) Uncropped Western blot images (showing LAMP2-A signals) are shown from three independent differentiation batches for individual iMG lines. The quantified LAMP2-A bands have been marked with arrows. Asterisks indicate cropped lanes (1, 2, 6, 8, 10, 13 from blot a) shown in Figure 4.

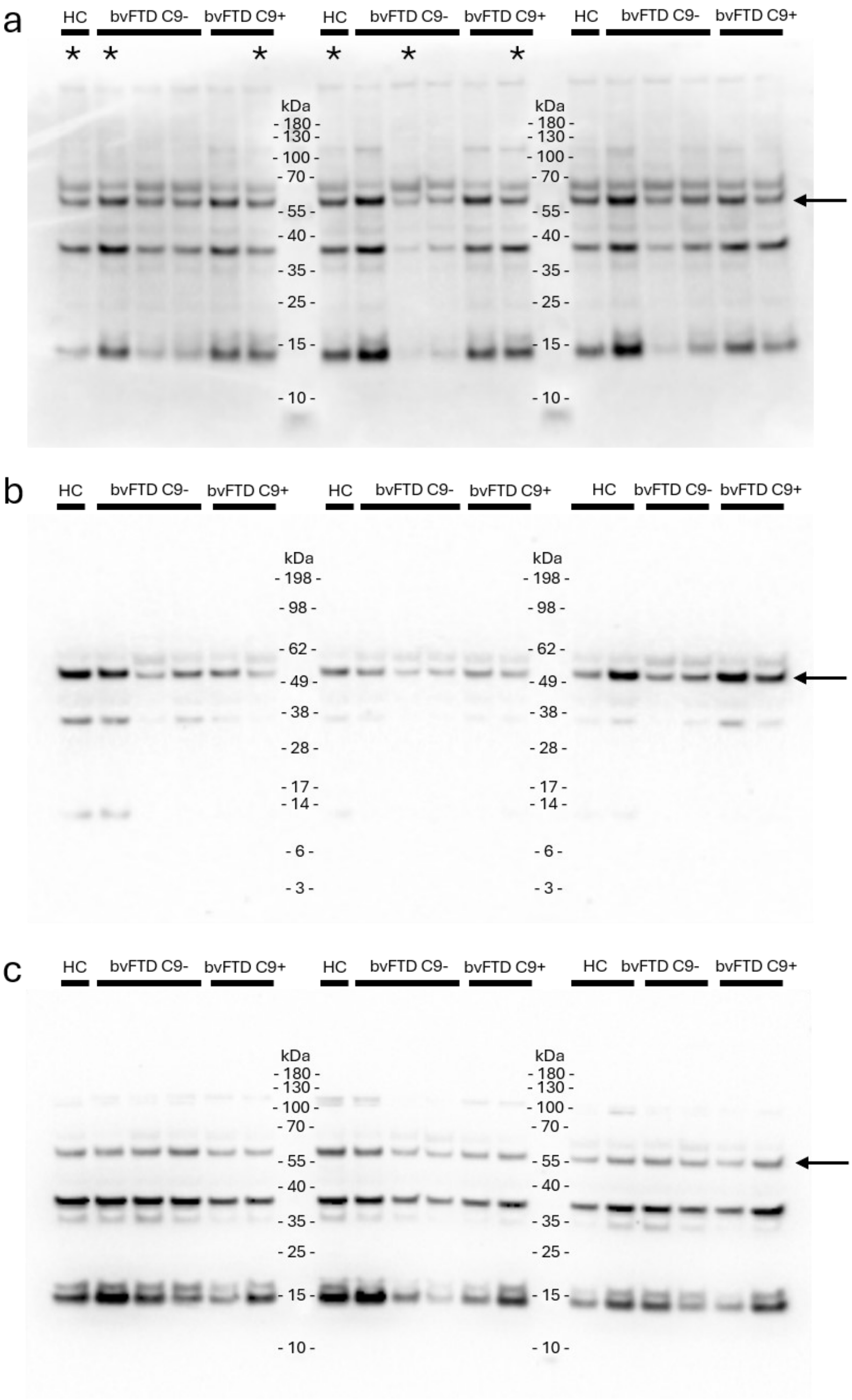

Figure S14. p62/SQSTM1 Western Blots from iMG of healthy controls, sporadic (C9-) and C9-HRE-carrying (C9+) bvFTD patients, related to Figure 4. (a-c) Uncropped Western blot images (showing p62/SQSTM1 signals) are shown from three independent differentiation batches for individual iMG lines. The quantified p62/SQSTM1 bands have been marked with arrows. Asterisks indicate cropped lanes (1, 2, 6, 8, 10, 13 from blot a) shown in Figure 4.

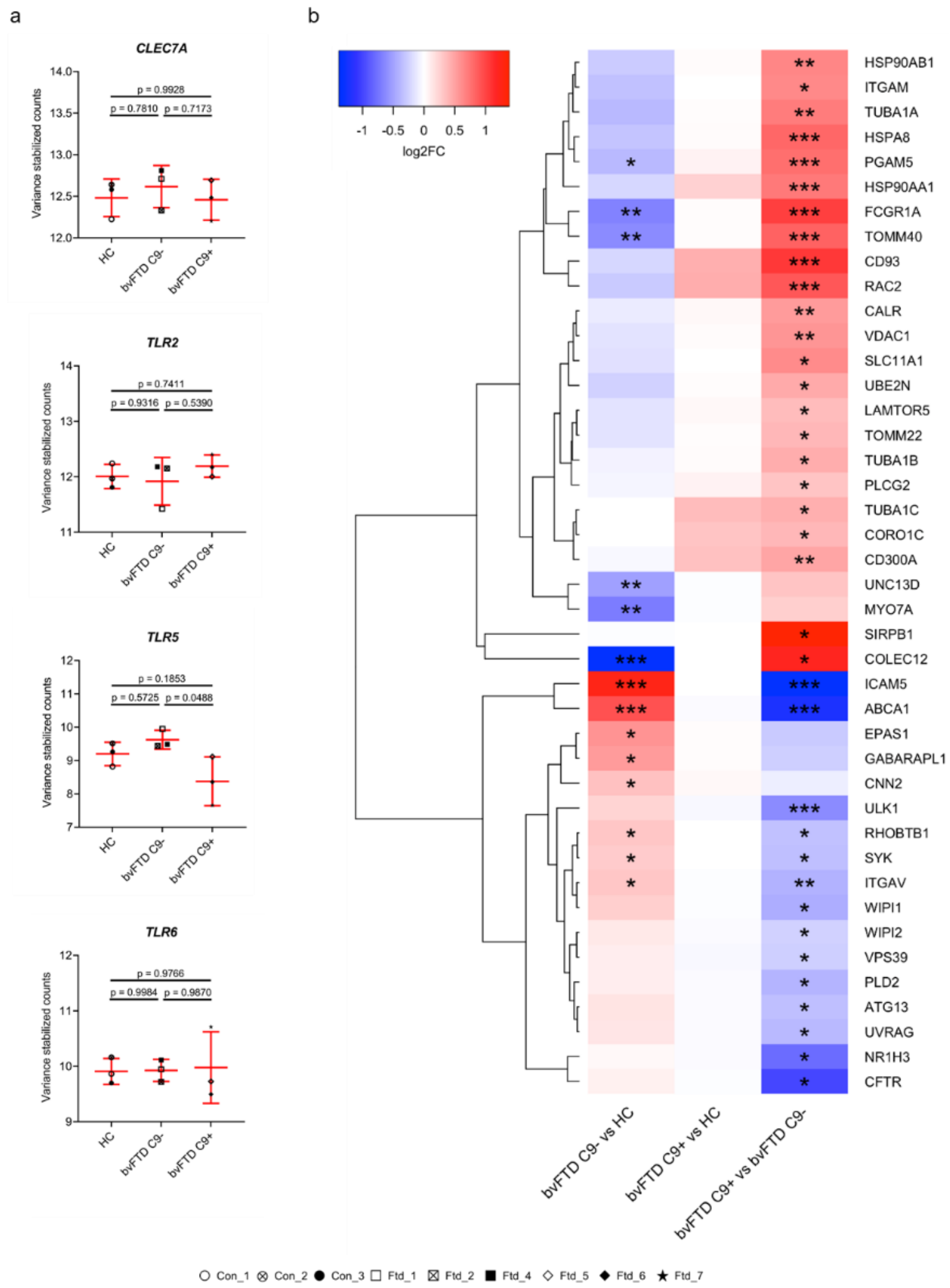

Figure S15. Phagocytosis-related gene expression in iMG of healthy controls, sporadic (C9-) and C9-HRE-carrying (C9+) bvFTD patients. (a) RNA levels according to RNA sequencing are shown for each donor as mean ± standard deviation of variance stabilized counts of *CLEC7A*: $F = 0.3732$, $p = 0.7035$; *TLR2*: $F = 0.6503$, $p = 0.5551$; *TLR5*: $F = 4.926$, $p = 0.0542$; *TLR6*: $F = 0.02323$, $p = 0.9771$; n [HC] = 3; n [bvFTD C9-] = 3; n [bvFTD C9+] = 3; each data point represents one RNA sample per donor (n). One-way ANOVA followed by Tukey's multiple comparisons test. (b) Heatmap shows the comparison-wise fold changes (log2) for all genes in the Gene ontology biological process phagocytosis category (GO 0006909), phagolysosome (GO 0032010), autophagosome-lysosome fusion (GO:0061909), and the Reactome category autophagy (R-HSA-9612973) that are differentially expressed (FDR < 0.05) in any of the three comparisons. Significance levels: * = 0.01 ≤ padj < 0.05, ** = 0.001 ≤ padj < 0.01, *** = padj < 0.001. Data are shown from one differentiation batch. The individual iMG lines are indicated by different symbols at the bottom.

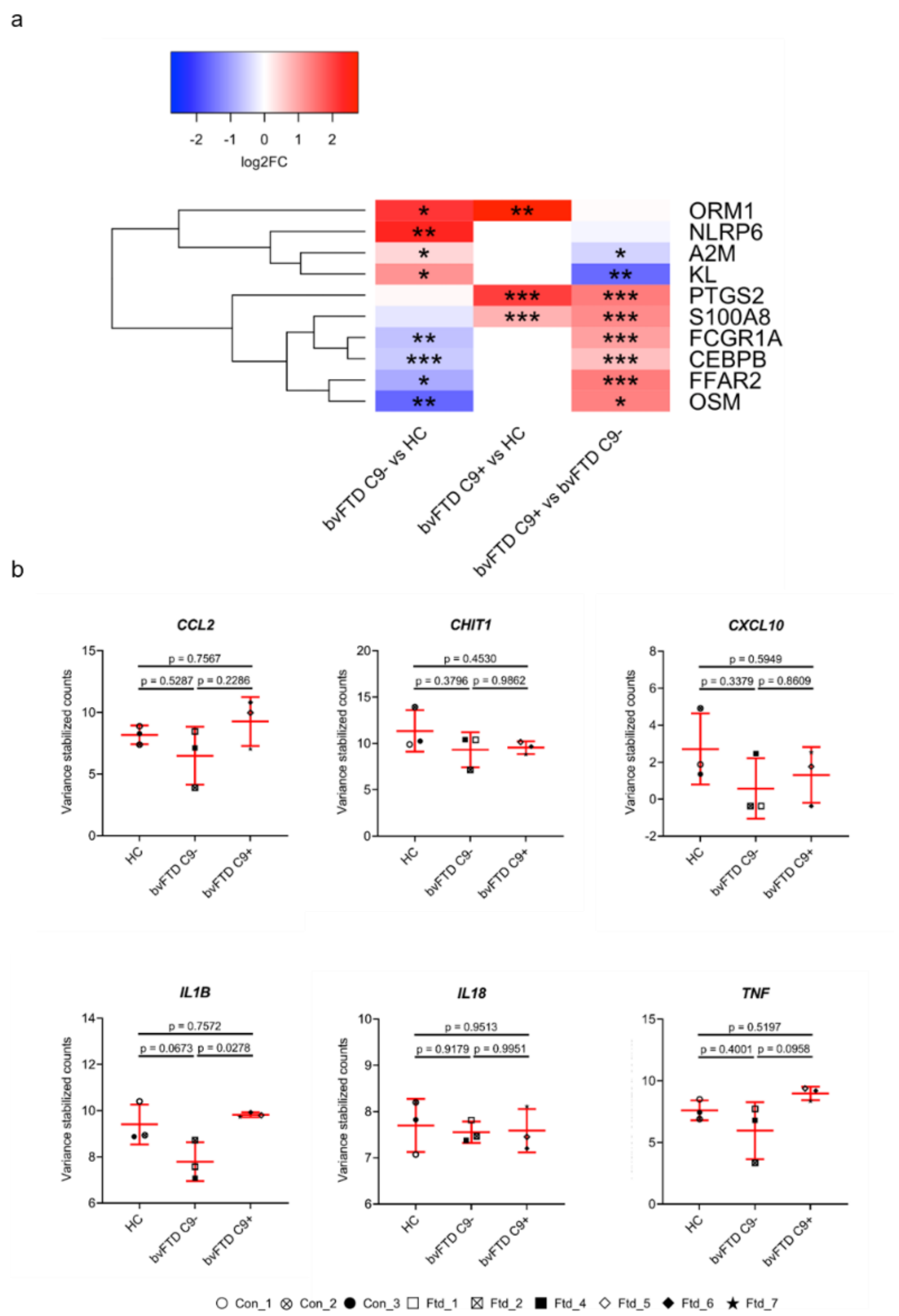

Figure S16. Inflammation-related gene expression in iMG of healthy controls, sporadic (C9-) and C9-HRE-carrying (C9+) bvFTD patients. (a) Heatmap shows the comparison-wise fold changes (log2) for all genes in the Gene ontology biological process acute inflammatory response (GO 0002526) that are differentially expressed (FDR < 0.05) in any of the three comparisons. (b) RNA levels of *CCL2*: F = 1.767, p = 0.2493; *CHIT1*: F = 1.252, p = 0.3511; *CXCL10*: F = 1.230, p = 0.3567; *ILB*: F = 7.063, p = 0.0265; *IL18*: F = 0.08600, p = 0.9187; *TNF*: F = 3.261, p = 0.1100. Data are shown for each donor as mean ± standard deviation of variance stabilized counts; each data point represents one RNA sample per donor (n); n [HC] = 3; n [bvFTD C9-] = 3; n [bvFTD C9+] = 3. Significance levels: * = 0.01 ≤ padj < 0.05, ** = 0.001 ≤ padj < 0.01, *** = padj < 0.001. Data are shown from one differentiation batch. The individual iMG lines are indicated by different symbols at the bottom.

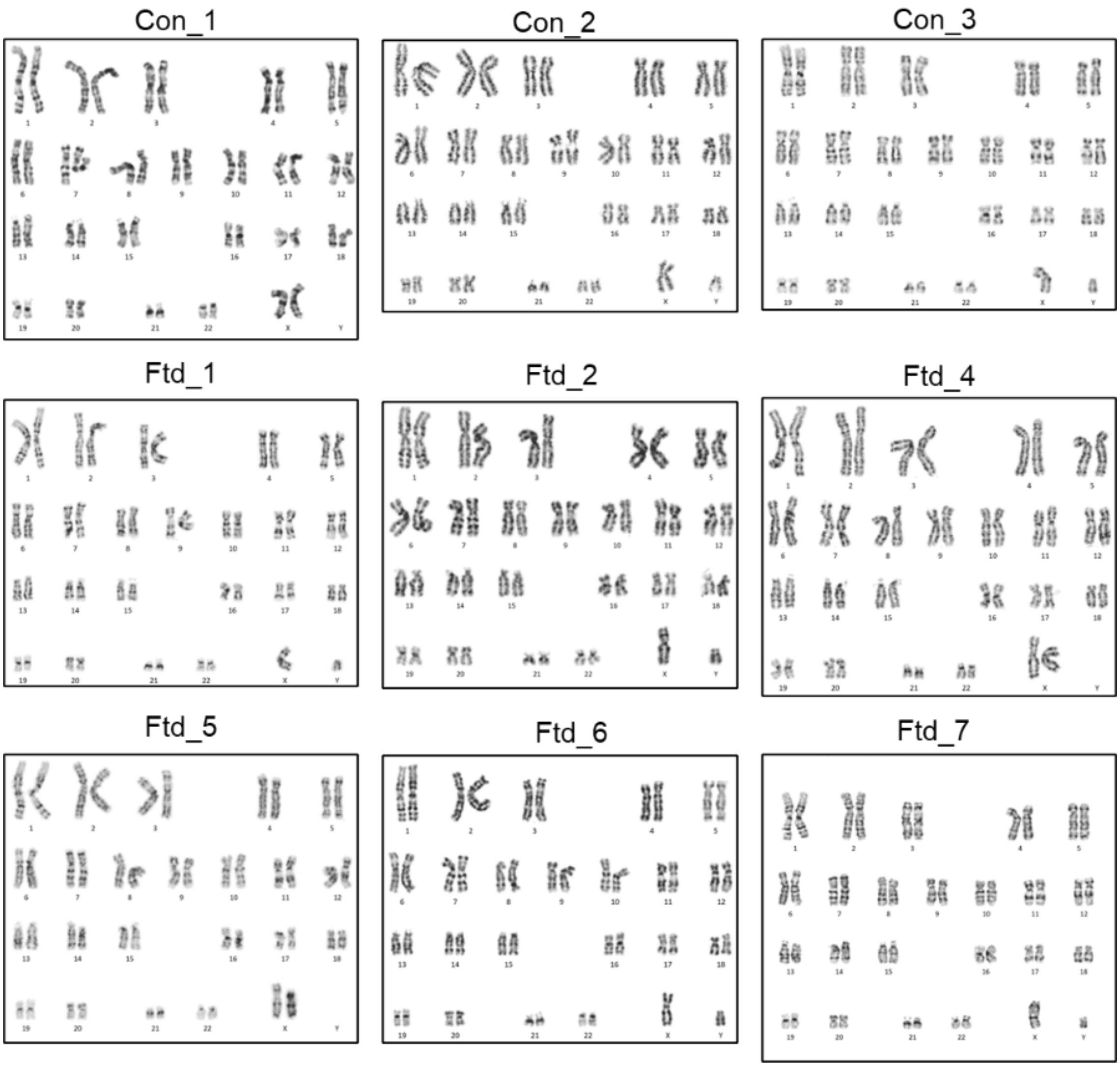

Figure S17. Karyotypes of iPSCs from healthy controls, sporadic (C9-) and C9-HRE-carrying bvFTD (C9+) patients. The karyotypes from all lines are shown. Karyotype analysis (p=21-38) by an experienced hospital geneticist indicated that two healthy control iPSC lines (Con_1; Con_2) had non-pathological chromosomal alterations (in one, structurally abnormal short arm of chromosome 12; and in the other, short arm was replaced with long arm of chromosome 20, thereby forming an isochromosome 20q). The other iPSC lines showed normal karyotypes.

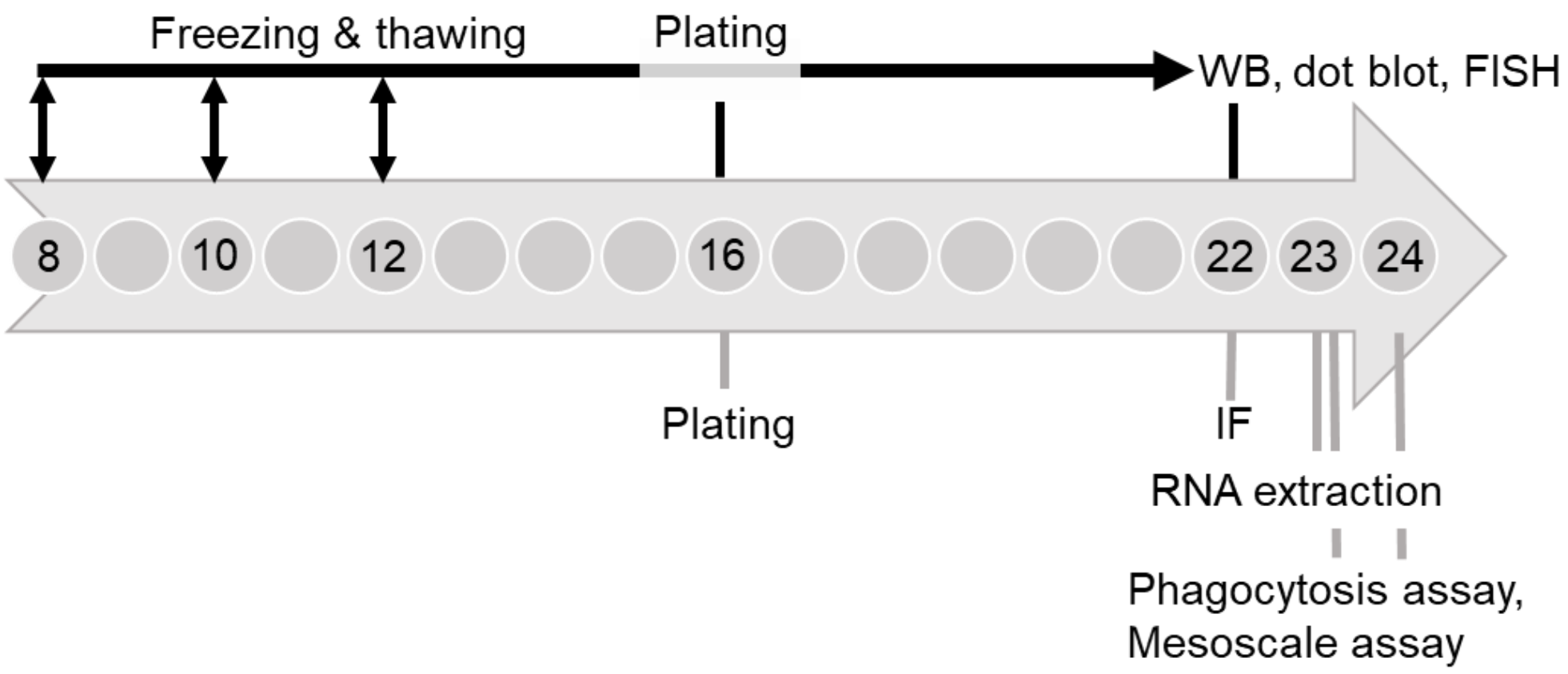

Figure S18. Timeline for iMG differentiation. Days of the differentiation protocol are indicated by the circles. Days of freezing and thawing of erythromyeloid progenitors and subsequent experiments with iMG are indicated in black. Experimental days with cells, which were not frozen and thawn in between are shown with bright gray lines.

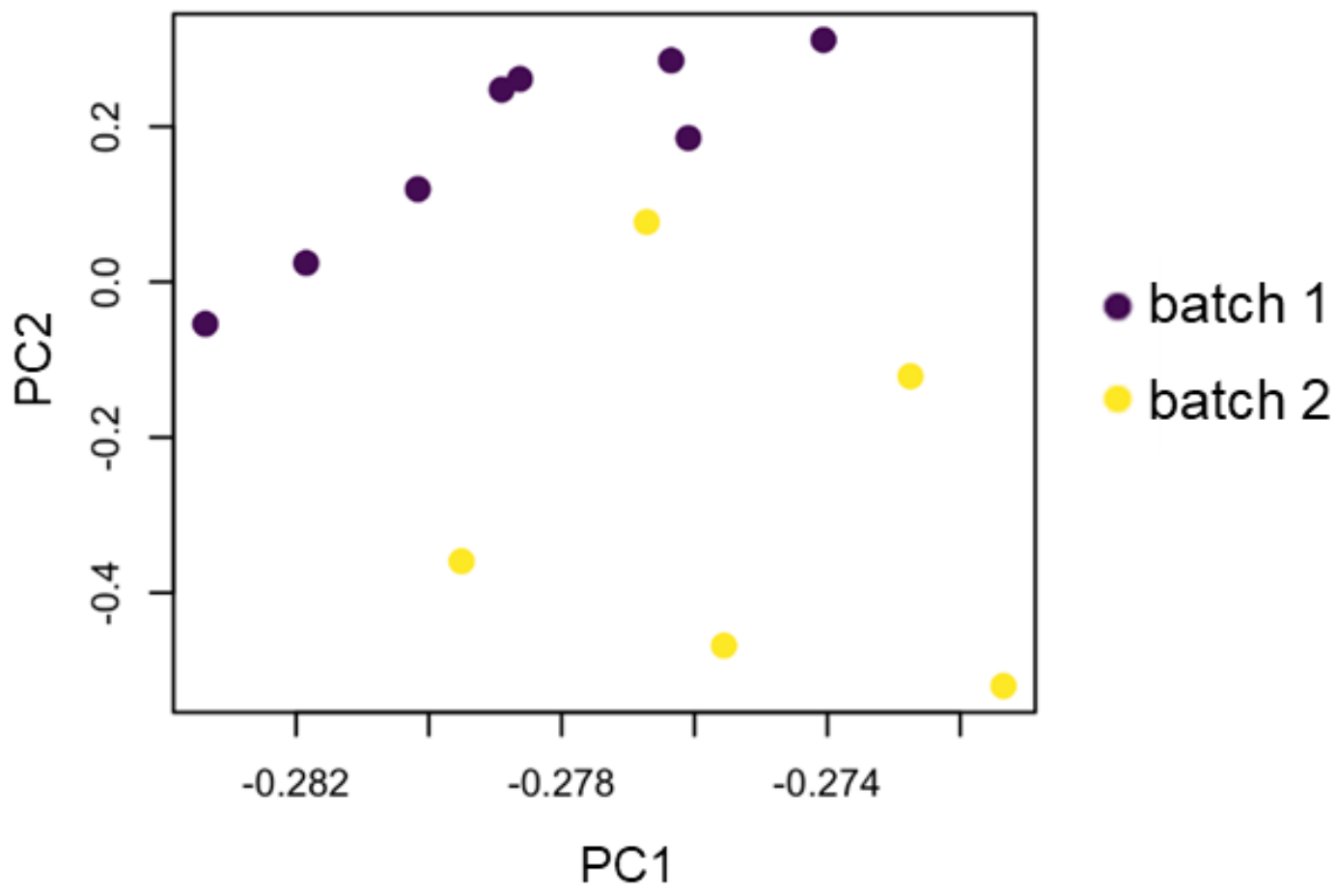

Figure S19. Principal component analysis of normalized RNA sequencing data. Principal component analysis revealed variation between sequencing batches to be corrected for in the differential expression analysis.

Supplemental Tables

Table S1. Information on donors; n. a. = not available

Sample ID	Sex	Age at sampling	Carriership of *C9orf72* hexanucleotide repeat expansion	Experiments in which the iMG were used	Passage at QC			
					Karyo-typing	PCR	IF	RP-PCR
Con_1	female	n.a.	no	all	25	12	12	-
Con_2	male	n.a.	no	all	26	11	11	-
Con_3	male	n.a.	no	all	38	12	13	-
Ftd_1	male	56	no	all	30	11	12	-
Ftd_2	male	67	no	all	17	10	11	-
Ftd_4	female	53	no	all	26	12	12	-
Ftd_5	female	55	yes	all	24	11	13	20
Ftd_6	male	77	yes	all	21	10	12	23
Ftd_7	male	50-54	yes	RNA sequencing, IF (C9orf72), Mesoscale, Western Blot (LC3B) FISH				16

Table S2. DEG and GSEA results on RNA sequencing data (s. online)

Supplemental Videos

Video S1. Phagocytosis assay of Con_1 line without prior serum starvation (s. online)

Video S2. Phagocytosis assay of Con_1 line after 24 h serum starvation (s. online)

Video S3. Phagocytosis assay of Con_2 line without prior serum starvation (s. online)

Video S4. Phagocytosis assay of Con_2 line after 24 h serum starvation (s. online)

Video S5. Phagocytosis assay of Con_3 line without prior serum starvation (s. online)

Video S6. Phagocytosis assay of Con_3 line after 24 h serum starvation (s. online)

Video S7. Phagocytosis assay of Ftd_1 line without prior serum starvation (s. online)

Video S8. Phagocytosis assay of Ftd_1 line after 24 h serum starvation (s. online)

Video S9. Phagocytosis assay of Ftd_2 line without prior serum starvation (s. online)

Video S10. Phagocytosis assay of Ftd_2 line after 24 h serum starvation (s. online)

Video S11. Phagocytosis assay of Ftd_4 line without prior serum starvation (s. online)

Video S12. Phagocytosis assay of Ftd_4 line after 24 h serum starvation (s. online)

Video S13. Phagocytosis assay of Ftd_5 line without prior serum starvation (s. online)

Video S14. Phagocytosis assay of Ftd_5 line after 24 h serum starvation (s. online)

Video S15. Phagocytosis assay of Ftd_6 line without prior serum starvation (s. online)

Video S16. Phagocytosis assay of Ftd_6 line after 24 h serum starvation (s. online)
